# Comparison of predictive tools for management of paediatric mild TBI: a prospective cohort study

**DOI:** 10.1016/j.eclinm.2025.103484

**Published:** 2025-09-09

**Authors:** Fredrik Wickbom, Sarah Thornberg, Jorge Sotoca Fernandez, Rasmus Silfver, Niklas Marklund, Johan Undén, The Scandinavian Neurotrauma Committee, Johan Ljungqvist, Anders Christian Feyling, David Nelson, András Buki, Mads Aarhus, Tor Brommeland, Ramona Åstrand, Johan Undén, Niklas Marklund, Teemu Luoto, Olga Calcagnile, Jussi Posti, Jens Jakob Riis, Eric Thelin, Elham Rostami, Fredrik Ginstman, Li Yang, Karoline Skogen, Shirin Kordasti

**Affiliations:** a Department of Clinical Sciences, Malmö, Lund University Faculty of Medicine, Lund, Sweden; b Department of Operation and Intensive Care, Halland Hospital Halmstad, Region Halland, Halmstad, Sweden; c Department of Paediatric Surgery, Queen Silvia Children's Hospital, Sahlgrenska University Hospital, Gothenburg, Sweden; d Paediatric Emergency Department, Department of Paediatrics, Skåne University Hospital, Malmö, Sweden; e Department of Surgery, Halland Hospital Varberg, Region Halland, Halland, Sweden; f Department of Clinical Sciences, Lund University, Lund, Sweden; g Department of Neurosurgery, Skåne University Hospital Lund, Lund, Sweden

**Keywords:** Guidelines, Traumatic brain injury, Children, Diagnostic accuracy, Comparison

## Abstract

**Background:**

Multiple clinical practice guidelines and head computed tomography decision rules exist for emergency department (ED) triage of children with traumatic brain injury (TBI). These vary in structure, aim, target cohort, and outcomes, yet are used clinically in similar populations. We compared important clinical and practical characteristics of all major guidelines in a large, real-world paediatric traumatic brain injury (TBI) cohort.

**Methods:**

Prospective, pragmatic, observational study of children (<18 years of age) presenting with mild-moderate TBI to 16 EDs in Sweden and Norway, including prospective documentation of guideline-specific risk factors and outcomes between April 2018 and May 2024. We assessed the diagnostic accuracy and characteristics of CATCH, CATCH2, CHALICE, PECARN, SNC16, PREDICT, and NICE23, both within guideline-specific application cohorts and across the full study cohort. The primary comparative outcome was significant trauma-related findings on cranial computed tomography (cCT). Secondary outcomes were neurosurgical interventions and guideline-specific endpoints. The study was registered at ClinicalTrials.gov (NCT05964764).

**Findings:**

The full cohort consisted of 3012 children (median age, 5.6 years; SD, 4.8). Among these, 0.9% (27/3012) had significant cCT findings, and 2/3012 (0.07%) required neurosurgery. CATCH and CATCH2 could be applied to 31.0% (934/3012) of patients, whereas the remaining guidelines were applicable to >94% of the cohort. In a comparative analysis concerning significant cCT findings, the lowest sensitivity estimates were 74.1% (95% CI: 53.7–88.9) for both PECARN ≥2 years and PREDICT ≥2 years; the highest was 100% (95% CI: 87.2–100.0) for SNC16. Specificity ranged from 41.6% (95% CI: 39.8–43.4) for SNC16 to 78.3% (95% CI: 76.8–79.8) for CHALICE. Mandatory cCT rates varied from 1.2% (PREDICT ≥2 years) to 29.9% (CATCH2).

**Interpretation:**

A head-to-head comparison in a real-world, paediatric, TBI cohort highlights key features of established guidelines and decision rules, offering insight into their comparative diagnostic accuracy, practical application and clinical impact.

**Funding:**

This work was supported by non-commercial state funding from Södra Sjukvårdsregionen, Vetenskapliga Rådet (Hallands Hospital), and 10.13039/501100009963Forskning och Utveckling Halland.

**Research in context**

**Evidence before this study**

We searched Medline, Scopus, CINAHL, and Cochrane Database of Systematic Reviews, without date or language restrictions, for prospective studies comparing diagnostic performance of at least two clinical decision rules and/or clinical practice guidelines in paediatric mild traumatic brain injury (TBI) with focus on emergency department triage for computed tomography (CT), observation and/or discharge.

The search terms used were: (TBI OR head injury OR head trauma) AND (children OR pediatric OR paediatric) AND (validation OR accuracy OR performance OR comparison) AND (guidelines OR decision rules) AND (prospective), including related words.

After removal of duplicates, 100 publications were retrieved, with one relevant study added after screening reference lists. Seven studies reported comparative performance of two or more predictive tools. None reported data for more than three rules. Performance of major guidelines such as PREDICT, NICE, and SNC have not been comparatively analysed.

**Added value of this study**

This is the first large, prospective study, based upon a real-life cohort and with rigorous mitigation of bias, examining the comparative effectiveness of all major guidelines for initial management of children with TBI. Despite assumed similarities of these predictive tools, this study illustrates important differences in diagnostic performance, practical application and clinical impact.

**Implications of all the available evidence**

Guidelines for management of children with TBI differ substantially in diagnostic performance, with variations in sensitivity, specificity, and the proportion of children recommended for CT imaging, observation, or discharge. Importantly, the variability in guideline performance observed in this study underscores the need for further comparative effectiveness research. Large, multicenter validation studies with harmonised methodologies are essential to determine the external validity of these guidelines across diverse healthcare settings.

## Introduction

Traumatic brain injury (TBI) in children constitutes a global public health challenge, with both acute and long-term consequence.^1^ The vast majority of cases (>90%) fall within the mild spectrum, with outcomes ranging from no sequelae to urgent need for neurosurgical evacuation of intracranial haematomas and/or persistent deficits across a wide range of cognitive functions.^2^^,^^3^ The primary focus during initial assessment in the emergency department (ED) is to evaluate the need for further investigations to detect intracranial complications.^2^ To support clinical decision-making, several clinical decision rules (CDRs) and practice guidelines (CPGs) have been developed over the past two decades.

A clinical decision rule (CDR) is defined as a tool derived from original research that incorporates three or more variables from patient history, physical examination, or simple tests.^4^ In contrast, a clinical practice guideline (CPG) consists of “systematically developed statements to assist practitioner and patient decisions about appropriate health care for specific clinical circumstances”. These guidelines are based on reviews of the best available evidence and may range from detailed algorithms to care pathways.^5^ The development of both CDRs and CPGs should be followed by structured evaluation and implementation processes, which are critical to their effectiveness in clinical practice.^4^^,^^5^ In this paper, both CPGs and CDRs are collectively referred to as “predictive tools”.

In a recent analysis, the Pediatric Emergency Care Applied Research Network (PECARN) guideline was the predictive tool to receive the highest grade, evaluated in 23 studies.^6^^,^^7^ The Children's Head Injury Algorithm for the Prediction of Important Clinical Events (CHALICE) was evaluated in 13 studies, and the Canadian Assessment of Tomography for Childhood Head Injury (CATCH) guideline in 11 studies.^6^^,^^8^^,^^9^ An updated version of the CATCH rule, CATCH2, was published in 2018, demonstrating improved sensitivities following prospective validation and refinement.^10^ More recently, the Australian and New Zealand Guideline for Mild to Moderate Head Injuries in Children (PREDICT) and the updated Head Injury: Assessment and Early Management guideline from the National Institute for Health and Care Excellence (NICE23) were published in 2021 and 2023, respectively.^11^^,^^12^ In the Nordic region, the Scandinavian guideline for the management of minor and moderate head trauma in children (SNC16)^2^ was published in 2016 by the Scandinavian Neurotrauma Committee, an independent association of medical specialists dedicated to advancing research, education, and clinical guidelines in the field of neurotrauma. These have recently been validated, indicating adequate performance.^13^

Direct comparisons of diagnostic accuracy for PECARN, CATCH, and CHALICE were initially performed in a single-centre, prospectively sampled cohort of 1009 children with minor head injuries by Easter et al., in 2014.^14^ The Australasian Paediatric Head Injury Rules Study (APHIRST) externally validated and compared the diagnostic performance of PECARN, CHALICE, and CATCH in a prospective multicenter cohort of 20,137 children with blunt head trauma.^15^ More recently, other predictive tools, including SNC16, have also been validated.^16^, ^17^, ^18^ Other systematic reviews have used alternative methods to analyse paediatric TBI guidelines.^19^

While these predictive tools may seem similar, they differ significantly in scope, objectives, target outcomes, processes for development, evaluation, and implementation.^6^^,^^19^^,^^20^ Few prospective, direct head-to-head comparisons have been conducted. In particular, significant tools such as NICE23, PREDICT, and SNC16 have not been comparatively analysed. In addition to diagnostic performance and applicability, the practical and clinical impact on the health care system and patients is also essential to evaluate.

The primary objective of this study is to compare the characteristics and diagnostic performance of all well-established international clinical predictive tools for paediatric TBI in a real-world cohort of children with head injury.

## Methods

### Study design and participants

Prospective, pragmatic, observational cohort study conducted at 16 emergency departments in Sweden and Norway. Patient enrolment took place between April 2018 and May 2024. Children under 18 years of age presenting with a recent (<24 h) blunt head trauma and a Glasgow Coma Scale (GCS) score of 9–15 were eligible for inclusion. Exclusion criteria included suspected child abuse, penetrating head injury, lack of informed consent, lack of a personal identification number, and concurrent enrolment in another study that could influence clinical management. Enrolment was performed by the treating clinician in the emergency department and study participation did not alter clinical management. Due to practical and clinical constraints, enrolment was non-consecutive, and screening logs could not be maintained across all centres due to logistical and data protection limitations. To evaluate the representativeness of the study sample, a controlled sampling period was conducted in spring 2023. Over 144 cumulative days across 8 of the 16 participating hospitals, all eligible patients—both enrolled and non-enrolled—were recorded. For each patient, data were collected on age, sex, SNC16 risk category, and time of ED presentation. Comparisons were made between included and missed patients during the controlled period, as well as between patients enrolled during this period and those enrolled in the rest of the Scandinavian cohort. The findings indicated that the included sample was representative of the broader target population.^13^ Further details regarding participating centres are provided in the Supplementary Table S2.

Overall study methodology and statistical considerations have been published elsewere.^21^ The study is registered at ClinicalTrials.gov (NCT05964764). We adhered to the Standards for the Reporting of Diagnostic Accuracy Studies (STARD) guidelines.

### Ethics

Ethical approval was obtained from the ethical review board in Lund, Sweden (EPN Lund Dnr 2017/238, with approved amendments EPN Lund Dnr 2018/670; EPN Lund Dnr 2020-05876; EPN Lund Dnr 2021-01580; EPN Lund Dnr 2022-01686-02; EPN Lund Dnr 2023-00412-02); and EPN Norway EPN Norway reference number 1085. Informed verbal consent was obtained from a caregiver, and from the child when deemed capable of understanding the study information.

### Procedures

Data used to classify diagnostic tests were prospectively recorded by the enrolling physician (or by a nurse if the patient was discharged without a physician's evaluation) at the time of the ED visit. Outcome data was collected by site investigators more than one-month post-trauma through review of medical records. Cranial computed tomography (cCT) findings were documented as reported by the on-call radiologist at each participating site, who was unaware of study enrolment. In Scandinavia, pathological CT findings are routinely assessed by a neurosurgeon and, if needed, by a neuroradiologist at a tertiary hospital.

We evaluated multiple clinical practice guidelines and decision rules, with tool specific risk factors and outcomes prospectively documented: CATCH,^9^ CATCH2,^10^ CHALICE,^8^ PECARN (<2 years),^7^ PECARN (≥2 years),^7^ SNC16,^2^ PREDICT (<2 years),^12^ PREDICT (≥2 years),^12^ and NICE23.^11^ Definitions used for variable coding, as well as the decision trees guiding management recommendations, are described in the protocol (Supplementary Tables S2–S6).^21^ For guidelines that use age-based dichotomisation (PECARN and PREDICT), results are presented separately for the <2-year and ≥2-year groups.

For each predictive tool, a “positive” test was defined as having one or more rule-specific risk factors. In predictive tools with varying levels of risk factors, we also present analyses based solely on high-risk criteria as Supplementary Data. In addition to each predictive tool's primary and secondary endpoints, we evaluated the outcomes “neurosurgery” and “significant cCT findings”. Neurosurgery was defined as any neurosurgical procedure or intervention (including neurointensive care with sedation, intubation, and controlled ventilation for non-surgical injuries, such as diffuse axonal injury) within one week of the trauma. Significant cCT findings were defined as any trauma-related intracranial injury detected on CT (<1-week post-trauma), including intracranial haemorrhages and cerebral contusions, but excluding linear skull fractures. Patients with isolated, non-depressed, linear fractures were not included in the comparative outcome as these patients do not require immediate intervention, although clinical follow-up may be suitable due to risk for development of growing skull fracture or cognitive issues.

### Statistics

Categorical variables are presented as counts and percentages. Prevalence of rule-specific predictors are provided descriptively. Application rates (i.e., the proportion of patients who met each predictive tool's inclusion criteria without exclusion criteria) were calculated for each of the nine predictive tools, defining nine corresponding tool-specific application cohorts. In addition, all 3012 patients were included in a comparison cohort, aiming to compare predictive tool performance and effect in this pragmatically sampled cohort.

For each predictive tool, point estimates (with 95% Clopper–Pearson confidence intervals; 95% CI) were calculated for sensitivity, specificity, positive predictive value (PPV), and negative predictive value (NPV) in both the application cohorts and the full comparison cohort. We also present estimated mandatory CT rates (i.e., the proportion of patients for whom immediate CT is recommended) for each predictive tool in both the application and comparison cohorts. For predictive tools offering observation as an alternative to immediate CT, we calculated the rates of these options. We further report a composite measure termed “any intervention”, defined as any active recommendation (CT, observation and/or admission) other than immediate discharge.

The missing data patterns in the Scandinavian cohort has been extensively evaluated in a previous publication, where only 3.65% (110/3012) of the patients had missing on one or more SNC16 predictors.^13^ In line with the approach taken in that publication after evaluation of a multiple imputation model, and in a similar comparative study,^15^ missing data were imputed as negative, which is the most clinically plausible assumption in this context.

All analyses were performed using IBM SPSS Statistics, version 30.0.

### Role of the funding source

This study was non-commercially funded by Södra Sjukvårdsregionen, Vetenskapliga rådet (Hallands Hospital), and Forskning och Utveckling Halland. No pharmaceutical company or external agency was involved in the development of the manuscript. The funders had no role in study design, data collection, data analysis, data interpretation, or writing of the report. The authors had full access to all the data in the study and had final responsibility for the decision to submit for publication.

## Results

3012 children aged 0–17 years (mean age 5.6 years, SD 4.8) were enrolled. Of these, 1770 (58.8%) were boys; 29.0% (873/3012) were younger than 2 years, and 14.0% (424/3012) were younger than 1 year at the time of injury. Falls were the most common mechanism of injury (68.0%, 2048/3012), and 96.2% (2898/3012) of patients presented with a GCS score of 15 in the emergency department. The overall cCT referral rate was 7.3% (219/3012). Of the 33 patients (1.1%) who had trauma-related findings on cCT, 27 (0.9% of the total cohort) were classified as having significant cCT findings; two patients (0.07%) required neurosurgical intervention, and no deaths were reported. Full cohort characteristics have been published previously,^13^ and demographic data are presented in Table 1. No children that were discharged from hospital showed any later complications regarding the pre-defined outcomes.

**Table 1:** Cohort characteristics.

Cohort characteristics	n (%)
**Age and sex (number of patients with valid data, when missing data occurs)**	
Mean age (years)	5.6 years (SD 4.8 years)
Age <1 year	424 (14.1%)
<2 years	873 (29.0%)
≥2 years	2139 (71.0%)
Boys	1770 (58.8%)
Girls	1242 (41.2%)
**Trauma mechanism**^a^	
Fall	2048 (68.0%)
Sports	358 (11.9%)
In traffic	217 (7.2%)
Head hits stationary object	196 (6.5%)
Hit by moving object (low speed)	55 (1.8%)
Head hit by projectile or object in high speed	51 (1.7%)
Run into/collided with another person	49 (1.6%)
Assault	19 (0.6%)
Unknown/Other mechanism	19 (0.6%)
**Clinical characteristics**	
Trauma alarm activated according to criteria for high velocity injury mechanisms	80 (2.7%)
GCS 9–13	24 (0.8%)
GCS 14	90 (3.0%)
GCS 15	2898 (96.2%)
Loss of consciousness (n = 3005)	380 (12.6%)
No LOC	2625 (87.2%)
<5 s	97 (3.2%)
5 s–1 min	172 (5.7%)
1–5 min	51 (1.7%)
>5 min	8 (0.3%)
Unknown	52 (1.7%)
Headache (n = 2996)	963 (32.0%)
Severe (n = 2996)	25 (0.8%)
Progressive (n = 2912)	81 (2.7%)
Vomiting (n = 2991)	821 (27.3%)
1 time	298 (9.9%)
2 times	196 (6.5%)
3 times	139 (4.6%)
4 or more times	172 (5.7%)
Abnormal behaviour according to guardian (n = 2892)	561 (18.6%)
Posttraumatic amnesia (n = 3001)	395 (13.1%)
Shunt	1 (<0.01%)
Scalp haematoma (n = 2998)	591 (19.6%)
Large (>3 cm) (n = 2996)	91 (3.0%)
Frontal (n = 2997)	343 (11.4%)
Parietal or temporal (n = 2997)	133 (4.5%)
Occipital (n = 2997)	114 (3.8%)
Clinical signs of skull base fracture (n = 3006)	9 (0.3%)
Depressed skull fracture	3 (0.1%)
Post-traumatic seizure (n = 2994)	25 (0.8%)
Focal neurological motor or sensory deficit (n = 2993)	25 (0.8%)
Abnormal pupils (n = 3000)	13 (0.4%)
Ataxia (n = 3004)	4 (0.1%)
Aphasia (n = 2999)	4 (0.1%)
Anticoagulation	1 (<0.1%)
Coagulation disorder	8 (0.3%)
Age <2 y and irritability	10 (0.3%)
Bulging fontanel (n = 2997)	0 (0%)
Multiple risk factors^b^	647 (21.5%)
**Outcomes**	
Cranial computed tomography	219 (7.3%)
Discharge from ED^c^	1938 (64.3%)
Prolonged observation in ED or ward^d^	868 (28.8%)
Admission to ward^c^	516 (17.1%)
Clinically important intracranial injury^e^	9 (0.3%)
Death	0 (0%)
Neurosurgery	2 (0.07%)
Admission to ward for 2 days or more due to head injury	9 (0.3%)
Intubation 1 day or more due to pathological traumatic CT findings	0 (0%)
cCT findings^f^	33 (1.1%)
Significant cCT findings^f^	27 (0.9%)

Age 0–17 years and <24 h since trauma. Continuous data are presented with mean and SD. Categorical variables are presented as number and percentages. Number of patients with missing data for these variables are presented in Supplementary Table S2.

Abbreviations: GCS = Glasgow Coma Scale (paediatric GCS was reported for patients <5 years of age); LOC = loss of consciousness; cCT = cranial computed tomography; ED = emergency department.

Adapted unchanged from Wickbom et al., 2025, Diagnostic accuracy of the Scandinavian guidelines for minor and moderate head trauma in children: a prospective, pragmatic, validation study. The Lancet Regional Health Europe. Distributed under CC BY 4.0 https://creativecommons.org/licenses/by/4.0/.

a There were eight prespecified options for reporting of the trauma mechanism, including a free text option. Free text answers were then re-categorised by FW and JU into the prespecified categories or into two new categories. These were 1) hit by moving object (low speed) and 2) Run into/collided with another person. Where the free text reported trauma mechanism was deemed impossible to recategorize into any of the categories, the trauma mechanism remained classified as “other”.

b More than one of the risk factors presented in the SNC16 guideline flowchart figure (12).

c As reported in medical records follow-up questionnaire.

d As reported by ED-physician or ED-nurse.

e Death, neurosurgery, admission to hospital ward 2 days or more due to head injury or intubation 1 day or more due to pathological traumatic CT findings.

f Significant CT findings are defined as a possibly trauma-related intracranial finding on CT scan, such as cranial fractures or acute intracranial haematoma, but not including undislocated skull fractures.

**Fig. 1:**
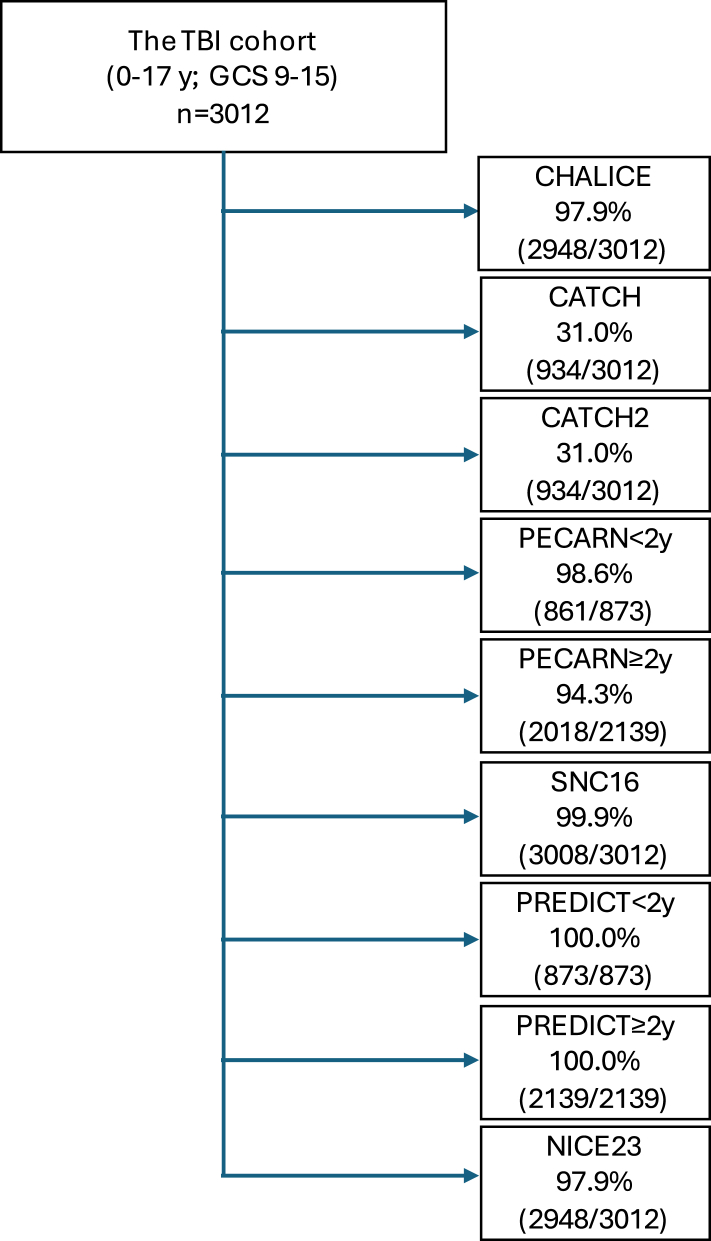
Flowchart describing cohort allocation to seven rule-specific application cohorts with resulting application rates for the guidelines and prediction rules explored in this study. Allocation is based on rule-specific inclusion and exclusion criteria. Patients in the original cohort (n = 3012) are comprising the comparison cohort. Abbreviations: TBI = traumatic brain injury; GCS = Glasgow Coma Scale; PECARN = Pediatric Emergency Care Applied Research Network guideline; CHALICE = The Children's Head Injury Algorithm for the Prediction of Important Clinical Events; CATCH = the Canadian Assessment of Tomography for Childhood Head Injury guideline; CATCH2 = the Canadian Assessment of Tomography for Childhood Head Injury guideline refined 8-item rule; PREDICT = Australian and New Zealand Guideline for Mild to Moderate Head Injuries in Children; NICE23 = Head Injury: Assessment and Early Management guideline from the National Institute for Health and Care Excellence 2023 version; SNC16 = the Scandinavian guideline for the management of minor and moderate head trauma in children.

Application rates varied from 31.0% (934/3012) for CATCH/CATCH2 to 100% (3012/3012) for PREDICT. All other predictive tools showed application rates between 94.3% and 99.9%, as illustrated in Fig. 1. Reasons for non-applicability of a predictive tool are reported in the Supplementary Table S3.

Table 2 presents the prevalence of rule-specific risk factors in each application cohort and in the comparison cohort. The number of patients who met the respective predictive tool's primary outcome ranged from 0.07% (2/3012) for SNC16, 0.1% (3/3012) for CATCH and CATCH2, 0.2% (5/3012) for PREDICT, 0.2% (6/3012) for PECARN, and up to 0.9% (26/3012) for CHALICE and NICE23.

**Table 2:** Prevalence of rule-specific risk factors in application cohorts and comparison cohort.

	Application cohort	Comparison cohort
**CATCH and CATCH2 predictors**		
GCS score <15 at 2 h after injury	43/934 (4.6%)	68/3012 (2.3%)
Suspected open or depressed skull fracture	0/934 (0.0%)	3/3012 (0.1%)
History of worsening headache	40/934 (4.3%)	81/3012 (2.7%)
Irritability on examination	10/934 (1.1%)	10/3012 (0.3%)
Any sign of basal skull fracture	3/934 (0.3%)	9/3012 (0.3%)
Large, boggy haematoma of the scalp	12/934 (1.3%)	52/3012 (1.7%)
Dangerous mechanism of injury	206/934 (22.1%)	636/3012 (21.1%)
≥4 episodes of vomiting^a^	160/934 (17.1%)	172/3012 (5.7%)
**SNC16 predictors**		
GCS 9–13	24/3008 (0.8%)	24/3012 (0.8%)
GCS 14	89/3008 (3.0%)	90/3012 (3.0%)
Focal neurological deficit	45/3008 (1.5%)	45/3012 (1.5%)
Post-traumatic seizure	25/3008 (0.8%)	25/3012 (0.8%)
Clinical signs of skull base fracture	9/3008 (0.3%)	9/3012 (0.3%)
Clinical signs of depressed skull fracture	3/3008 (0.1%)	3/3012 (0.1%)
LOC (suspected/brief)	380/3008 (12.6%)	380/3012 (12.6%)
LOC (>1 min)	59/3008 (2.0%)	59/3012 (2.0%)
Anticoagulation	1/3008 (0.0%)	1/3012 (0.0%)
Coagulation disorder	8/3008 (0.3%)	8/3012 (0.3%)
Post-traumatic amnesia	394/3008 (13.1%)	395/3012 (13.1%)
Severe headache	25/3008 (0.8%)	25/3012 (0.8%)
Progressive headache	80/3008 (2.7%)	81/3012 (2.7%)
Abnormal behaviour according to guardian	559/3008 (18.6%)	561/3012 (18.6%)
Vomiting ≥2	506/3008 (16.8%)	507/3012 (16.8%)
Shunt	1/3008 (0.0%)	1/3012 (0.0%)
Age <2 years and irritability	10/3008 (0.3%)	10/3012 (0.3%)
Age <2 years and large, temporal or parietal scalp haematoma	12/3008 (0.4%)	12/3012 (0.4%)
Age <1 year	424/3008 (14.1%)	424/3012 (14.1%)
Bulging fontanel	0/3008 (0.0%)	0/3012 (0.0%)
Activated trauma alarm in ED	79/3008 (2.6%)	80/3012 (2.7%)
**CHALICE predictors**		
Witnessed loss of consciousness >5 min	8/2948 (0.3%)	8/3012 (0.3%)
History of amnesia >5 min	149/2948 (5.1%)	155/3012 (5.1%)
Abnormal drowsiness	63/2948 (2.1%)	64/3012 (2.1%)
≥3 vomiting episodes after head injury	308/2948 (10.4%)	311/3012 (10.3%)
Suspicion of non-accidental injury^b^	0/2948 (0.0%)	0/3012 (0.0%)
Seizure after head injury	25/2948 (0.8%)	25/3012 (0.8%)
GCS score <14 or GCS <15 if aged <1 year	34/2948 (1.2%)	34/3012 (1.1%)
Suspicion of penetrating^b^ or depressed skull fracture	3/2948 (0.1%)	3/3012 (0.1%)
Suspicion tense fontanelle	0/2948 (0.0%)	0/3012 (0.0%)
Signs of basal skull fracture	8/2948 (0.3%)	9/3012 (0.3%)
Positive focal neurology	44/2948 (1.5%)	45/3012 (1.5%)
Bruise, swelling, or laceration >5 cm if aged <1 year	53/2948 (1.8%)	53/3012 (1.8%)
High-speed RTA as pedestrian, cyclist, or vehicle occupant	4/2948 (0.1%)	4/3012 (0.1%)
Fall >3 m	14/2948 (0.5%)	14/3012 (0.5%)
High-speed injury from a projectile or an object	68/2948 (2.3%)	69/3012 (2.3%)
**PECARN predictors (age <2 years)**		
GCS < 15	24/861 (2.8%)	114/3012 (3.8%)
Other signs of altered mental status	6/861 (0.7%)	91/3012 (3.0%)
Palpable or unclear skull fracture	11/861 (1.3%)	24/3012 (0.8%)
Scalp haematoma (occipital, parietal, or temporal)	77/861 (8.9%)	247/3012 (8.2%)
History of loss of consciousness for ≥5 s	34/861 (3.9%)	231/3012 (7.7%)
Severe mechanism of injury (traffic)	0/861 (0.0%)	20/861 (0.7%)
Severe mechanism of injury (fall)	190/861 (22.1%)	519/3012 (17.2%)
Severe mechanism of injury (high impact object)	14/861 (1.6%)	69/3012 (2.3%)
Acting abnormally per parent report	145/861 (16.8%)	561/3012 (18.6%)
**PECARN predictors (age ≥2 years)**		
GCS < 15	57/2018 (2.8%)	114/3012 (3.8%)
Other signs of altered mental status	57/2018 (2.8%)	90/3012 (3.0%)
Signs of basilar skull fracture	8/2018 (0.4%)	9/3012 (0.3%)
History of loss of consciousness	297/2018 (14.7%)	380/3012 (12.6%)
History of vomiting	533/2018 (26.4%)	821/3012 (27.3%)
Severe mechanism of injury (traffic)	20/2018 (1.0%)	20/3012 (0.7%)
Severe mechanism of injury (fall)	94/2018 (4.7%)	130/3012 (4.3%)
Severe mechanism of injury (high impact object)	54/2018 (2.7%)	69/3012 (2.3%)
Severe headache	20/2018 (1.0%)	25/3012 (0.8%)
**PREDICT predictors (age <2 years)**		
GCS 9–13	2/873 (0.2%)	24/3012 (0.8%)
GCS 14	25/873 (2.9%)	90/3012 (3.0%)
Other signs of altered mental status	9/873 (1.0%)	91/3012 (3.0%)
Abnormal neurological examination	6/873 (0.7%)	45/3012 (1.5%)
Severe mechanism of injury (traffic)	0/873 (0.0%)	20/3012 (0.7%)
Severe mechanism of injury (fall)	195/873 (22.3%)	519/3012 (17.2%)
Severe mechanism of injury (high impact object)	14/873 (1.6%)	69/3012 (2.3%)
Post-traumatic seizure(s)	9/873 (1.0%)	25/3012 (0.8%)
Palpable skull fracture	11/873 (1.3%)	24/3012 (0.8%)
Non-frontal scalp haematoma	77/873 (8.8%)	247/3012 (8.2%)
History of LOC ≥5 s	34/873 (3.9%)	231/3012 (7.7%)
Acting abnormally per parent	148/873 (17.0%)	561/3012 (18.6%)
<6 months old	120/873 (13.7%)	120/3012 (4.0%)
Neurodevelopmental disorders	1/873 (0.1%)	11/3012 (0.4%)
Ventricular shunt	1/873 (0.1%)	1/3012 (<0.1%)
Bleeding disorders	3/873 (0.3%)	9/3012 (0.3%)
Possible abusive head trauma^b^	0/873 (0.0%)	0/3012 (0.3%)
**PREDICT predictors (age ≥2 years)**		
GCS 9–13	22/2139 (1.0%)	24/3012 (0.8%)
GCS 14	65/2139 (3.0%)	90/3012 (3.0%)
Other signs of altered mental status	82/2139 (3.8%)	91/3012 (3.0%)
Abnormal neurological examination	39/2139 (1.8%)	45/3012 (1.5%)
Severe mechanism of injury (traffic)	20/2139 (0.9%)	20/3012 (0.7%)
Severe mechanism of injury (fall)	101/2139 (4.7%)	130/3012 (4.3%)
Severe mechanism of injury (high impact object)	55/2139 (2.6%)	69/3012 (2.3%)
Post-traumatic seizure(s)	16/2139 (0.7%)	25/3012 (0.8%)
Signs of skull base fracture	9/2139 (0.4%)	9/3012 (0.3%)
History of LOC	325/2139 (15.2%)	380/3012 (12.6%)
History of vomiting	575/2139 (26.9%)	821/3012 (27.3%)
Severe headache	25/2139 (1.2%)	25/3012 (0.8%)
Neurodevelopmental disorders	10/2139 (0.5%)	11/3012 (0.4%)
Ventricular shunt	0/2139 (0.0%)	1/3012 (<0.1%)
Bleeding disorders	6/2139 (0.3%)	9/3012 (0.3%)
Possible abusive head trauma^b^	0/2139 (0.0%)	0/3012 (0.3%)
**NICE23 predictors**		
Suspicion of non-accidental injury^b^	0/2948 (0.0%)	0/3012 (0.0%)
Post-traumatic seizure but no history of epilepsy	25/2948 (0.8%)	25/3012 (0.8%)
GCS score <14 or GCS <15 if aged <1 year	34/2948 (1.2%)	34/3012 (1.1%)
GCS score of less than 15 at 2 h after the injury	67/2948 (2.3%)	68/3012 (2.3%)
Suspicion of penetrating^b^ or depressed skull fracture	3/2948 (0.1%)	3/3012 (0.1%)
Suspicion tense fontanelle	0/2948 (0.0%)	0/3012 (0.0%)
Signs of basal skull fracture	8/2948 (0.3%)	9/3012 (0.3%)
Positive focal neurology	44/2948 (1.5%)	45/3012 (1.5%)
Bruise, swelling, or laceration >5 cm if aged <1 year	53/2948 (1.8%)	53/3012 (1.8%)
Witnessed loss of consciousness >5 min	8/2948 (0.3%)	8/3012 (0.3%)
Any bleeding or clotting disorders (liver failure, haemophilia, taking anticoagulants or antiplatelets)^c^	8/2948 (0.3%)	8/3012 (0.3%)
Abnormal drowsiness	63/2948 (2.1%)	64/3012 (2.1%)
3 or more discrete episodes of vomiting	308/2948 (10.4%)	311/3012 (10.3%)
High-speed RTA as pedestrian, cyclist, or vehicle occupant	4/2948 (0.1%)	4/3012 (0.1%)
Fall >3 m	14/2948 (0.5%)	14/3012 (0.5%)
High-speed injury from a projectile or an object	68/2948 (2.3%)	69/3012 (2.3%)
History of amnesia >5 min	149/2948 (5.1%)	155/3012 (5.1%)

Abbreviations: GCS = Glasgow Coma Scale; RTA = road traffic accident; LOC = loss of consciousness; ED = emergency department; PECARN = Pediatric Emergency Care Applied Research Network guideline; CHALICE = The Children's Head Injury Algorithm for the Prediction of Important Clinical Events; CATCH = the Canadian Assessment of Tomography for Childhood Head Injury guideline; CATCH2 = the Canadian Assessment of Tomography for Childhood Head Injury guideline refined 8-item rule; PREDICT = Australian and New Zealand Guideline for Mild to Moderate Head Injuries in Children; NICE23 = Head Injury: Assessment and Early Management guideline from the National Institute for Health and Care Excellence 2023 version; SNC16 = the Scandinavian guideline for the management of minor and moderate head trauma in children.

a CATCH2 predictor.

b Exclusion criteria for enrolment.

c Aspirin not included.

In the comparative analysis, point estimates for sensitivity ranged from 74.1% (95% CI: 53.7–88.9) for both PECARN ≥2 years and PREDICT ≥2 years, to 100% (95% CI: 87.2–100.0) for SNC16 (see Table 3). Specificity was lowest for SNC16 (41.6%, 95% CI: 39.8–43.4) and highest for CHALICE (78.3%, 95% CI: 76.8–79.8). All predictive tools demonstrated low positive predictive values (<3.4%) and high negative predictive values (>99.5%). All predictive tools except PECARN ≥2 years and PREDICT ≥2 years achieved 100% sensitivity for detecting the need for neurosurgery (Supplementary Table S4).

**Table 3:** Diagnostic accuracy to predict significant cCT findings in comparison cohort.

	Sensitivity % (CI 95)	Specificity % (CI 95)	PPV % (CI 95)	NPV % (CI 95)
CHALICE	81.5% (61.9–93.7)	78.3% (76.8–79.8)	3.3% (2.1–4.9)	99.8% (99.5–99.9)
CATCH	77.8% (57.7–91.4)	74.4% (72.8–76.0)	2.7% (1.7–4.1)	99.7% (99.4–99.9)
CATCH2	85.2% (66.3–95.8)	70.6% (68.9–72.2)	2.6% (1.6–3.8)	99.8% (99.5–99.9)
PECARN < 2 years	85.2% (66.3–95.8)	55.3% (53.5–57.1)	1.7% (1.1–2.5)	99.8% (99.4–99.9)
PECARN ≥ 2 years	74.1% (53.7–88.9)	55.8% (54.0–57.6)	1.5% (0.9–2.3)	99.6% (99.1–99.8)
SNC16	100.0% (87.2–100.0)	41.6% (39.8–43.4)	1.5% (1.0–2.2)	100.0% (99.7–100.0)
PREDICT < 2 years	85.2% (66.3–95.8)	51.8% (50.0–53.6)	1.6% (1.0–2.4)	99.7% (99.3–99.9)
PREDICT ≥ 2 years	74.1% (53.7–88.9)	54.6% (52.8–56.4)	1.5% (0.9–2.2)	99.6% (99.1–99.8)
NICE23	81.5% (61.9–93.7)	77.4% (75.9–78.9)	3.2% (2.0–4.7)	99.8% (99.5–99.9)

Data presented when applying all risk predictors in respective predictive tool.

Abbreviations: cCT = cranial computed tomography; PPV = positive predictive value; NPV = negative predictive value; PECARN = Pediatric Emergency Care Applied Research Network guideline; CHALICE = The Children's Head Injury Algorithm for the Prediction of Important Clinical Events; CATCH = the Canadian Assessment of Tomography for Childhood Head Injury guideline; CATCH2 = the Canadian Assessment of Tomography for Childhood Head Injury guideline refined 8-item rule; PREDICT = Australian and New Zealand Guideline for Mild to Moderate Head Injuries in Children; NICE23 = Head Injury: Assessment and Early Management guideline from the National Institute for Health and Care Excellence 2023 version; SNC16 = the Scandinavian guideline for the management of minor and moderate head trauma in children.

Patients with significant cCT findings in the comparison cohort, missed by a predictive tool, are shown in Table 4. Both PECARN ≥2 years and PREDICT ≥2 years missed a 25-month-old girl with a palpable skull fracture, with significant cCT findings and need for neurosurgery due to a depressed skull fracture.

**Table 4:** Missed patients with significant cCT findings^b^ in comparison cohort (n = 3012) for each predictive tool.

ID^a^	CT findings	Age	Sex	Trauma mechanism	GCS	Clinical data and risk factors	Missed by guideline^c^	Neurosurgery
1	Depressed skull fracture (<1 bone width), small haematoma, unclear if subdural or epidural.	0	Male	Fall	15	Previously healthy, no medications, fall 1–1.5 m, headache (unspecified), scalp haematoma (medium size, parietal, boggy).	**CHALICE** *PECARN ≥ 2 y* *PREDICT ≥ 2 y* **NICE23**	No
2	Depressed skull fracture (>1 bone width), haematoma under fracture.	2	Female	Fall	15	Previously healthy, no medications, fell backward into wooden bench, clinical signs of palpable and depressed skull fracture.	**PECARN ≥ 2 y** **PREDICT ≥ 2 y**	Yes
3	Depressed skull fracture (<1 bone width), extracranial haematoma.	0	Male	Fall	15	Previously healthy, no medications, fall <1 m, abnormal behaviour according to guardian, scalp haematoma (large, parietal, firm).	**CATCH** **CATCH2** *PECARN ≥ 2 y* *PREDICT ≥ 2 y*	No
7	Skull base fracture.	1	Male	Fall	14	Previously healthy, no medications, fall 1.6–3 m, suspected LOC: 5 s–1 min, scalp haematoma (medium size, frontal), affected orientation/mental status (agitation and irritability).	**CHALICE** **NICE23**	No
8	Skull base fracture.	0	Male	Fall	15	Previously health, other unspecified medication, fall <1 m, abnormal behaviour according to guardian, clinical signs of palpable skull fracture, scalp haematoma (large, parietal, boggy).	*PECARN ≥ 2 y* *PREDICT ≥ 2 y*	No
10	Depressed skull fracture (>1 bone width), skull base fracture.	5	Male	In traffic	15	Previously healthy, no medications, single-vehicle accident in unspecified motorised vehicle, 21–30 km/h, helmet, signs of cervical spine injury, trauma alarm.	**CHALICE** *PECARN < 2 y* **PECARN ≥ 2 y** *PREDICT < 2 y* **PREDICT ≥ 2 y** **NICE23**	No
11	Contusions.	11	Female	In traffic	15	Previously healthy, no medications, single-vehicle bicycle accident at 21–30 km/h, no helmet, headache (moderate, unchanged), vomit 3 times, laceration on the scalp.	**CATCH** **CATCH2** *PECARN < 2 y* *PREDICT < 2 y*	No
19	Skull base fracture.	0	Male	Fall	15	Previously healthy, no medications, fall <1 m, scalp haematoma (large, parietal, boggy).	*PECARN ≥ 2 y* *PREDICT ≥ 2 y*	No
20	Traumatic subarachnoid haemorrhage.	17	Female	Fall	15	ADHD, stimulant medications for ADHD, fell of a sled and crashed into a pole, no helmet, <5 min of post-traumatic amnesia, suspected LOC of unclear duration, scalp laceration, trauma alarm.	**CATCH** **CATCH2** **CHALICE** *PECARN < 2 y* *PREDICT < 2 y* **NICE23**	No
21	Skull base fracture, acute subdural haematoma, contusions, depressed fracture (<1 bone width).	1	Male	Fall	15	Previously healthy, no medications, fall <1 m, scalp haematoma (large, parietal).	**CATCH** **CATCH2** **CHALICE** *PECARN ≥ 2 y* *PREDICT ≥ 2 y* **NICE23**	No
22	Linear skull fracture, skull base fracture.	2	Male	Hit by moving object	14	Previously healthy, no medications, the head was struck by a heavy door that fell over the child, small fractures below clavicles, trauma alarm, abnormal behaviour according to guardian, witnessed LOC 5 s–1 min, 4 or more episodes of vomiting, signs of fractures in the face, lacerations on the scalp, affected orientation/mental status (somnolence, disorientation), GCS 15 at 2 h post trauma.	**CATCH**	No
24	Linear skull fracture, epidural haematoma.	2	Male	Fall	15	Previously healthy, no medications, fall <1 m, 4 or more episodes of vomiting.	**CATCH** *PECARN < 2 y* *PREDICT < 2 y*	No

Abbreviations: cCT = cranial computed tomography; ID = identifier number; GCS = Glasgow Coma Scale; LOC = loss of consciousness; ADHD = attention deficit hyperactivity disorder; PECARN = Pediatric Emergency Care Applied Research Network guideline; CHALICE = The Children's Head Injury Algorithm for the Prediction of Important Clinical Events; CATCH = the Canadian Assessment of Tomography for Childhood Head Injury guideline; CATCH2 = the Canadian Assessment of Tomography for Childhood Head Injury guideline refined 8-item rule; PREDICT = Australian and New Zealand Guideline for Mild to Moderate Head Injuries in Children; NICE23 = Head Injury: Assessment and Early Management guideline from the National Institute for Health and Care Excellence 2023 version; SNC16 = the Scandinavian guideline for the management of minor and moderate head trauma in children.

a ID corresponds to number in previous publication were all significant cCT findings were reported.^13^

b Significant cCT findings are defined as a possibly trauma related intracranial finding on CT scan, such as cranial fractures or acute intracranial haemorrhage, but not including undislocated skull fractures.

c Missed patients per guideline: CATCH = 6; CATCH2 = 4; CHALICE = 5; SNC16 = 0; PECARN < 2 y = 4; PECARN ≥ 2 y = 7; PREDICT < 2 y = 4; PREDICT ≥ 2 y = 7; NICE23 = 5. Patients missed by a predictive tool outside age dichotomisation (over and under 2 years) are marked in *italic* and missed within age dichotomisation marked **bold**.

When evaluating diagnostic performance in each predictive tool's specific application cohort, point estimates for sensitivity to detect rule-specific primary endpoints were 100% (with wide confidence intervals) for SNC16 (n = 2), PREDICT <2 years (n = 1), and PECARN <2 years (n = 1). CHALICE (n = 24), NICE23 (n = 24), PECARN ≥2 years (n = 5), and PREDICT ≥2 years (n = 4) had sensitivity estimates of 75.0–83.3%, all with wide confidence intervals, and did not identify all patients meeting their respective primary outcomes. Due to inclusion restrictions in CATCH/CATCH2, three patients who met the rule-specific primary endpoint (two of whom required neurosurgery) were never assessed by the rule. Further details for each predictive tool are shown in the Supplementary Tables S5–S13 and summarised in Supplementary Table S14.

The predictive tools also differed in their recommended interventions for predictor-positive patients. Some stratified risk factors into multiple levels—mandating cCT, offering optional cCT or observation, or recommending observation alone. Across the entire cohort, mandatory cCT rates ranged from 1.2% (PREDICT ≥2 years) to 29.9% (CATCH2). Table 5 displays the total recommended interventions for each predictive tool, including a composite measure of “any intervention”. This composite measure varied substantially, from 22 to 23% for CHALICE and CATCH up to 59% for SNC16. CT and observation rates in tool-specific application cohorts are detailed in Supplementary Table S15.

**Table 5:** CT and observation rates in the comparison cohort.

	Mandatory cCT % (CI 95)	Optional cCT or observation % (CI 95)	Observation % (CI 95)	Sum (any intervention) % (CI 95)
CHALICE	22.2% (20.8–23.7)			22.2% (20.8–23.7)
CATCH	26.1% (24.5–27.7)			26.1% (24.5–27.7)
CATCH2	29.9% (28.3–31.5)			29.9% (28.3–31.5)
PECARN < 2 years	5.7% (4.9–6.6)	39.4% (37.6–41.1)		45.1% (43.3–46.9)
PECARN ≥ 2 years	5.3% (4.5–6.1)	39.1% (37.4–40.9)		44.4% (42.7–46.2)
SNC16	3.4% (2.8–4.0)	19.5% (18.1–21.0)	35.9% (34.2–37.6)	58.8% (57.0–60.5)
PREDICT < 2 years	1.7% (1.2–2.2)	13.2% (12.0–14.5)	33.6% (32.0–35.3)	48.5% (46.7–50.3)
PREDICT ≥ 2 years	1.2% (0.8–1.6)	8.4% (7.4–9.4)	36.1% (34.4–37.8)	45.7% (43.9–47.4)
NICE23	8.4% (7.4–9.4)		14.7% (13.5–16.0)	23.1% (21.6–24.6)

Data presented when applying all risk predictors in respective predictive tool.

Abbreviations: cCT = cranial computed tomography; PECARN = Pediatric Emergency Care Applied Research Network guideline; CHALICE = The Children's Head Injury Algorithm for the Prediction of Important Clinical Events; CATCH = the Canadian Assessment of Tomography for Childhood Head Injury guideline; CATCH2 = the Canadian Assessment of Tomography for Childhood Head Injury guideline refined 8-item rule; PREDICT = Australian and New Zealand Guideline for Mild to Moderate Head Injuries in Children; NICE23 = Head Injury: Assessment and Early Management guideline from the National Institute for Health and Care Excellence 2023 version; SNC16 = the Scandinavian guideline for the management of minor and moderate head trauma in children.

**Fig. 2:**
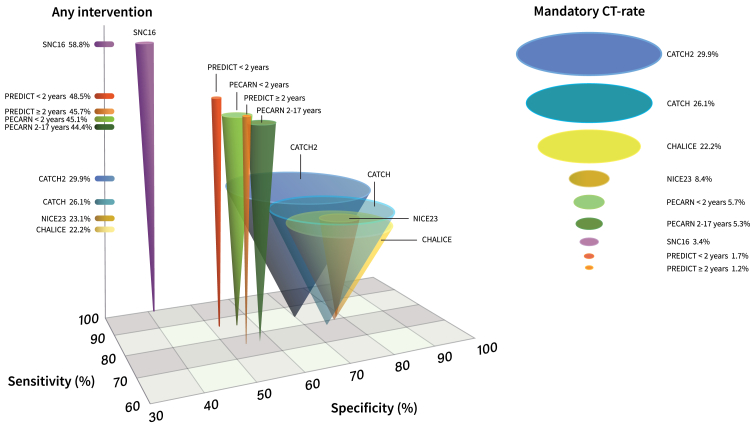
Sensitivity, specificity, any intervention rate, and mandatory cCT rate for all predictive tools assessed, based on comparative analysis in the full cohort (n = 3012) against the outcome of significant cCT findings. The figure displays nine inverted cones, each representing a predictive tool; PECARN and PREDICT are split by age group, resulting in two cones for each. The x-axis represents specificity, and the z-axis represents sensitivity; the tip of each cone marks the intersection between these two parameters. The height of each cone reflects the total intervention rate (i.e., proportion of patients recommended for either observation or cCT), with taller cones indicating a greater resource use to achieve a given diagnostic performance. The base diameter represents the mandatory cCT rate—a wider cone base indicates that more CT scans are mandated by the tool. Intervention rates (%) are shown on the y-axis, and mandatory cCT rates (%) are listed to the right of the figure, colour-matched to each cone. Abbreviations: cCT = cranial computed tomography; PECARN = Pediatric Emergency Care Applied Research Network guideline; CHALICE = The Children's Head Injury Algorithm for the Prediction of Important Clinical Events; CATCH = the Canadian Assessment of Tomography for Childhood Head Injury guideline; CATCH2 = the Canadian Assessment of Tomography for Childhood Head Injury guideline refined 8-item rule; PREDICT = Australian and New Zealand Guideline for Mild to Moderate Head Injuries in Children; NICE23 = Head Injury: Assessment and Early Management guideline from the National Institute for Health and Care Excellence 2023 version; SNC16 = the Scandinavian guideline for the management of minor and moderate head trauma in children.

Fig. 2 illustrates the diagnostic performance and clinical impact of each predictive tool using a three-dimensional cone model, where the tip indicates the sensitivity/specificity intersection, cone height reflects the total intervention rate (observation and/or cCT), and base diameter represents the mandatory cCT rate.

## Discussion

In this study, we applied all established clinical decision rules and practice guidelines to a prospective, real-world cohort of children with TBI and compared key diagnostic performance parameters, applicability and clinical impact. Because head-to-head comparisons can be complicated, we assessed performance in both the full cohort and in rule-specific application cohorts. In the full cohort, point sensitivity for detecting significant findings on cCT ranged from 74% for PREDICT (≥2 years) and PECARN (≥2 years) to 100% for SNC16, though with overlapping confidence intervals. Specificity in this same group was lowest for SNC16 (41.6%) and highest for CHALICE, CATCH, CATCH2, and NICE23 (ranging from 70.6% to 78.3%), with intermediate values for PREDICT and PECARN (51.8%–55.8%), showing significant differences between these three groups.

Clinical decision rules and guidelines are defined by their specific inclusion and exclusion criteria, which delineate the populations for which they were designed and validated. Applying a tool outside of its intended scope introduces uncertainty regarding diagnostic performance, as prior validation results may not be generalisable to other patient groups. However, in routine practice, clinicians are likely to apply predictive tools to all children presenting with head trauma at their institution. Therefore, application rates are an important measure of clinical relevance.^22^ The application rate for CATCH/CATCH2 was low (31.0%), which was expected given the tool's inclusion restrictions, comparable to 25% when evaluated in the APHIRST cohort.^15^ By contrast, the application rate for the remaining predictive tools was unexpectedly high (>94%), particularly for PECARN. In the APHIRST study—which included patients across all severities of TBI—the PECARN rule was applicable to only 75% of patients younger than 2 years and 76% of patients 2 years and older, whereas in our cohort (where no patients were excluded due to a trivial injury mechanism), 99% of patients <2 years of age and 94% of patients ≥2 years were eligible. Due to the restrictive inclusion criteria for CATCH/CATCH2, the rule failed to include three patients who met the rule-specific primary endpoint (i.e., need for neurological intervention) within its application cohort. This represents a major disadvantage and a potentially dangerous oversight. Moreover, not all patients meeting the rule-specific secondary outcome were identified in the application cohort, resulting in intermediate sensitivity (72.7%; 95% CI: 39.0–94.0) and specificity (71.6%; 95% CI: 68.6–74.5).

Both PECARN ≥2 years and PREDICT ≥2 years failed to identify one of two patients who required neurosurgery. The 25-month-old patient was positive for a risk factor included in the <2-year pathway for both PECARN and PREDICT, raising the question of whether strictly dichotomising by age is the best approach in clinical decision aids. Because children develop at different rates, age-based dichotomisation poses a risk, especially if clinicians are inexperienced and adhere rigidly to guidelines. The PECARN rule was meticulously developed using recursive partitioning, with a predefined age separation at two years, largely based on younger children's greater radiosensitivity, lower capacity to communicate, different trauma mechanisms and TBI risks.^7^ In the original derivation cohort, palpable skull fractures were present in 3.4% of children under two years of age and 2.1% of those aged two years and older. The PREDICT guideline is evidence- and consensus-based, similar to SNC16 and NICE23, influenced heavily by the PECARN rule, given its prominence in the literature.^2^^,^^6^^,^^11^^,^^12^ SNC16 and NICE23 do not dichotomise by age but instead categorise both signs of skull fracture and signs of basilar skull fracture as high-risk criteria. Thus, what began as relatively straightforward science (i.e., identifying specific risk factors for a prespecified outcome) has evolved into the realm of complex interventional research.^23^ We believe that future guideline development, evaluation, and refinement in paediatric mTBI should more strongly emphasise implementation aspects, a need already noted by guideline developers in both our and other settings.^24^^,^^25^

Newer predictive tools have placed increased emphasis on observation as an alternative management approach, given that it is both safe and cost-effective in reducing cCT use.^26^^,^^27^ These tools present a low mandatory cCT rate—1.2% for PREDICT ≥2 years, 1.7% for PREDICT <2 years, 3.4% for SNC16, 5.3% for PECARN ≥2 years, and 5.7% for PECARN <2 years—whereas CHALICE, CATCH, and CATCH2 have significantly higher mandatory cCT rates (22.2%, 26.1%, and 29.9%, respectively). They also remain the only rules that dichotomise patients strictly into “cCT” or “no cCT”. Interestingly, NICE23 presents users with two main interventions (mandatory cCT or observation) and omits the “optional cCT or observation” pathway seen in PECARN. Although NICE23 partly relies on CHALICE risk factors, this ongoing development of the NICE guidelines have decreased the mandatory cCT rate to 8.4%, which is still higher than that of SNC16, PECARN, and PREDICT. Given that some tools recommend CT in as few as 1.2% of patients, the question of optimal CT rate may be examined. While the principle of ALARA (As Low As Reasonably Achievable) remains foundational in paediatric imaging due to stochastic radiation risks, a CT rate of zero is neither feasible nor clinically desirable. Advances in CT technology have led to significant reductions in effective radiation dose, potentially altering the risk-benefit calculus of imaging.^28^ Future guideline development could benefit from closer collaboration with radiology societies, to help redefine acceptable imaging thresholds in light of evolving technical capabilities and resource constraints.

Despite the low mandatory cCT rate in PECARN, nearly 4 out of 10 children fall into the intermediate group, where clinicians can choose between optional cCT or observation. Although the PECARN rule is principally designed to identify children who do not need cCT, its flow-chart structure places responsibility on the user, potentially leading to a higher-than-necessary proportion of cCT referrals. We also calculated the total interventions recommended by each guideline (i.e., the number of patients for whom the guideline recommends an action other than discharge). Applying SNC16 leads to nearly 6 in 10 patients requiring some intervention, raising the question of whether the guideline is too conservatively designed, potentially elevating resource consumption. The actual clinical impact of implementing SNC16 remains unclear, as it calculates recommended clinical observation time from the time of trauma, which may imply that the recommended duration of observation has already passed by the time of assessment in the ED (hence, a proportion of these children may be discharged directly from the ED). Efforts to improve the specificity of high-resource-use tools—without compromising safety—are warranted. In adult TBI, the incorporation of biomarkers has shown promise in improving triage accuracy, and similar approaches are currently being explored in paediatric populations.^29^, ^30^, ^31^ Delayed presentation of intracranial haemorrhage beyond 6 h post-trauma is rare, and both PREDICT and NICE23 have adopted a 4-h reassessment after the head injury, which also serves as a pragmatic approach to reducing unnecessary observation time and overall resource consumption.^11^^,^^12^^,^^32^

In this study, we report key diagnostic performance parameters, applicability, and clinical impact for widely used guidelines and decision rules for triage of children with head injuries. As illustrated in Fig. 2, the classic trade-off between sensitivity and specificity is evident. Adoption of a clinical predictive tool must therefore be guided by this balance: if the priority is to detect all important outcomes, a greater proportion of children will require CT imaging or observation. In such contexts, the SNC16 guideline may be a suitable choice. It demonstrated 100% sensitivity, a low mandatory CT rate (3.4%), and high applicability to nearly all children presenting to the ED, without relying on age dichotomisation—which, based on our findings, may introduce risk near the threshold. However, the trade-off is higher resource use, as many patients are likely to require prolonged observation. Implementation of SNC16 may be challenging in small hospitals, particularly those lacking CT capability or overnight observation units. In rural settings—such as ski resorts or remote clinics—adherence may be impractical or impossible.

In contrast, when local resources are limited, a tool with higher specificity may be more appropriate, even if this comes at the cost of lower sensitivity. The NICE23 guideline is particularly relevant in such settings. It demonstrated intermediate specificity (77.4%), intermediate sensitivity for detecting significant CT findings (81.5%), and did not miss any patients who required neurosurgical intervention. One of NICE23's major advantages is its relatively low mandatory CT rate (8.4%), closely mirroring the actual CT rate observed in our cohort (7.3%). It also had a low overall intervention rate (23.1%).

In summary, none of the predictive tools assessed in this study fulfilled all desirable criteria: 100% sensitivity, high specificity, low CT and observation rates, high applicability, and ease of use. However, our head-to-head comparison highlights the respective strengths and limitations of each tool—information that is essential for clinical decision-making and highly valuable in designing the next generation of predictive tools for paediatric mTBI management in emergency settings.

While this study did not primarily aim to evaluate real-time clinical management,^21^ we acknowledge the importance of understanding how predictive tools align with actual practice, including observation strategies and delayed complications. Due to the pragmatic, multicentre design, clinical decisions—such as whether to observe a patient without imaging—were not standardised and likely varied across sites and over time. Although ED questionnaires captured the intended management plan at the time of enrolment, clinical trajectories may have changed (e.g., due to shift transitions or evolving symptoms). Therefore, outcomes were determined through structured medical record review, including whether complications emerged during the index visit or upon return. These management patterns were not analysed in the present manuscript but will be addressed in a forthcoming study exploring concordance between tool classification and real-world management.

Nevertheless, in our prior validation of the SNC16 guideline, several patients classified as low risk were found to have significant CT findings or required admission, although none required neurosurgical intervention.^13^ These findings underscore the need for further research on the clinical implementation and downstream consequences of observation-based strategies.

We acknowledge several additional limitations in our study. First, performing a head-to-head comparison in the full cohort potentially violates the intended application rules, predictor variables, and outcomes for each predictive tool. Moreover, each tool defines its own endpoints, which are heterogeneous, thereby reducing the ability to compare them and evaluate their effects. To mitigate this issue, we chose significant cCT findings as our primary comparative outcome—a compromise that offered sufficient prevalence for meaningful comparison, while remaining clinically relevant. We did not include isolated linear skull fractures in this outcome definition, as most tools are designed to detect complications requiring emergency intervention. It is also likely that clinicians do not strictly adhere to intended application criteria in everyday practice. Therefore, we argue that pragmatic comparisons in prospective, real-world cohorts remain highly valuable for both clinical and research purposes.^22^^,^^33^ Another limitation is the non-consecutive nature of enrolment. However, a controlled sampling period with parallel data collection on included and non-included patients indicated that the study sample was representative of the target population.^13^ Additionally, the number of patients meeting classical endpoints such as neurosurgery (n = 2) or clinically important TBI (n = 6) were low. We speculate that a possible reason is that the TBI entity has evolved over the years, possibly due to changes in injury mechanism and improvements in health care. We suggest that guidelines need to reflect these changes. It would be beneficial to adapt universal patient-important outcomes for both clinical guidelines and research, to harmonise efforts towards efficient and safe patient management.^1^^,^^20^

## Contributors

Fredrik Wickbom and Johan Undén conceived and designed the study, and obtained ethical approvals. Sarah Thornberg, Jorge Sotoca Fernandez, Rasmus Silfver, and Fredrik Wickbom coordinated patient enrolment and medical record follow-up across participating centres. Fredrik Wickbom curated the data and performed the statistical analyses. Both Fredrik Wickbom and Johan Undén accessed and verified the underlying data. Data interpretation was carried out by Fredrik Wickbom and Johan Undén, with critical input from Niklas Marklund, who also provided senior supervision together with Johan Undén. Fredrik Wickbom and Johan Undén conducted the literature search for the Research in Context panel and drafted the initial version of the manuscript, which was critically reviewed and revised by Sarah Thornberg, Jorge Sotoca Fernandez, Rasmus Silfver, and Niklas Marklund. The Scandinavian Neurotrauma Committee provided expert input during the study planning phase and facilitated clinical collaboration across sites. All authors read and approved the final version of the manuscript.

## Data sharing statement

Pseudonymised datasets will be available on reasonable request from the corresponding author for 10 years following the end of patient inclusion.

## Declaration of interests

All authors have completed the ICMJE uniform disclosure form at www.icmje.org/disclosure-of-interest/ and declare: No support from any organisation for the submitted work; no financial relationships with any organisations that might have an interest in the submitted work in the previous three years. JU and NM are unpaid board members of the Scandinavian Neurotrauma Committee, a non-profit organisation independent from financial company support, who are responsible for the SNC16 guideline. JU participated in the development of the SNC16 guideline. NM reports previous consulting fees from PolarCool Inc, current consulting fees from Teqcool Inc and honoraria from Balt Inc, with none of these having any connection with the present study, and is an unpaid board member of the European Neurotrauma Organisation, Swedish Sports Concussion Society and the European Association of Neurosurgical Societies. NM is also European Editor for J Neurotrauma.
