# Supplementary material for: Comparison of predictive tools for management of paediatric mild TBI: a prospective cohort study
Source: eClinicalMedicine. 2025 Sep 9;88:103484. doi: 10.1016/j.eclinm.2025.103484 (PMC12447897; doi:10.1016/j.eclinm.2025.103484)
Supplement: Supplementary Tables S1–S15 [file mmc1.pdf]

Supplementary online material for:

Comparison of predictive tools for management of paediatric mild TBI: a prospective cohort study

August 1, 2025

Contents list

Page	Content	Heading
3	Table S1	Scandinavian Neurotrauma Committee group member list
4	Table S2	Recruiting center characteristics
5	Table S3	Reasons for non-applicability of a predictive tool
6	Table S4	Diagnostic accuracy to predict neurosurgery in comparison cohort
7	Table S5	Diagnostic accuracy: CATCH2
8	Table S6	Diagnostic accuracy: CATCH
9	Table S7	Diagnostic accuracy: SNC16
10	Table S8	Diagnostic accuracy: CHALICE
11	Table S9	Diagnostic accuracy: PECARN < 2 years
12	Table S10	Diagnostic accuracy: PECARN ≥ 2 years
13	Table S11	Diagnostic accuracy: PREDICT < 2 years
14	Table S12	Diagnostic accuracy: PREDICT ≥ 2 years
15	Table S13	Diagnostic accuracy: NICE23
16	Table S14	Diagnostic accuracy to predict rule-specific primary endpoint in application cohorts
16	Table S15	CT and observation rates in the application cohorts

Supplementary Table S1. Scandinavian Neurotrauma Committee group member list

First names	Surnames
Johan	Ljungqvist
Anders Christian	Feyling
David	Nelson
András	Buki
Mads	Aarhus
Tor	Brommeland
Ramona	Åstrand
Johan	Undén
Niklas	Marklund
Teemu	Luoto
Olga	Calcagnile
Jussi	Posti
Jens Jakob	Riis
Eric	Thelin
Elham	Rostami
Fredrik	Ginstman
Li	Yang
Karoline	Skogen
Shirin	Kordasti

Supplementary Table S2. Recruiting center characteristics

	Hospital	Size	Included patients n (%)
**Sweden**			
1	Queen Silvia Children's Hospital, Sahlgrenska University Hospital, Gothenburg, Sweden	University ED	371 (12·3%)
2	Astrid Lindgren´s Childrens hospital, Solna, Stockholm, Sweden	University ED	298 (9·9%)
3	Skåne University Hospital, Lund, Sweden	University ED	365 (12·1%)
4	Skåne University Hospital, Malmö, Sweden	University ED	163 (5·4%)
5	Norrland University Hospital, Umeå, Sweden	University ED	257 (8·5%)
6	Örebro University Hospital, Region Örebro, Örebro, Sweden	University ED	9 (0·3%)
7	Halland Hospital Halmstad, Region Halland, Halland, Sweden	Regional ED	583 (19·4%)
8	Halland Hospital Varberg, Region Halland, Halland, Sweden	Regional ED	241 (8·0%)
9	Ryhov Hospital, Region Jönköpings län, Jönköping, Sweden	Regional ED	125 (4·2%)
10	Norra Älvsborgs Hospital, NU-sjukvården, Region Västra Götaland, Trollhättan, Sweden	Regional ED	137 (4·6%)
11	Mälarsjukhuset i Eskilstuna, Region Sörmland, Eskilstuna, Sweden	Regional ED	20 (0·7%)
12	Alingsås Hospital, Region Västra Götaland, Alingsås, Sweden	Local ED	158 (5·2%)
13	Mora Hospital, Region Dalarna, Mora, Sweden	Local ED	55 (1·8%)
14	Ystad Hospital, Region Skåne, Ystad, Sweden	Local ED	64 (2·1%)
15	Ljungby Hospital, Region Kronoberg, Ljungby, Sweden	Local ED	45 (1·5%)
**Norway**			
16	Haukeland University Hospital, Haukeland, Bergen, Norway	University ED	121 (4·0%)

Adapted unchanged from Wickbom et al., 2025, Diagnostic accuracy of the Scandinavian guidelines for minor and moderate head trauma in children: a prospective, pragmatic, validation study. The Lancet Regional Health Europe. Distributed under CC BY 4.0 https://creativecommons.org/licenses/by/4.0/

Supplementary Table S3. Reasons for non-applicability of a predictive tool

		Number of patients (%)
**Application rate**	**CATCH/CATCH2**	**934 (31·0%)**
Inclusion criteria	Minor head injury §	961 (31·9%)
Exclusion criteria	Head injury secondary to suspected child abuse*	0 (0·0%)
Exclusion criteria	Chronic generalized developmental delay	11 (0·4%)
Exclusion criteria	Acute focal neurologic deficit	45 (1·5%)
Exclusion criteria	Pregnant	1 (0·0%)
Exclusion criteria	Depressed fracture	3 (0·1%)
Exclusion criteria	Penetrating skull injury*	0 (0·0%)
Exclusion criteria	Returning for reassessment of a previously treated head injury*	0 (0·0%)

		Number of patients (%)
**Application rate**	**CHALICE**	**2948 (97·9%)**
Inclusion criteria	History or signs of injury to the head	3012 (100%)
Exclusion criteria	Age <16 years	64 (2·1%)

		Number of patients (%)
**Application rate**	**PECARN < 2 years**	**861 (98·6%)**
Inclusion criteria	Age < 2 years	873 (29·0%)
Exclusion criteria	Pre-existing neurological disorders complicating assessment	3 (0·3%)
Exclusion criteria	Trivial injury mechanism	0 (0·0%)
Exclusion criteria	Bleeding disorder	3 (0·3%)
Exclusion criteria	GCS 9-13	2 (0·2%)
Exclusion criteria	Known brain tumours	0 (0·0%)
Exclusion criteria	Neuroimaging at an outside hospital before transfer	5 (0·6%)
Exclusion criteria	Ventricular shunts	1 (0·1%)
Exclusion criteria	Penetrating trauma*	0 (0·0%)

		Number of patients (%)
**Application rate**	**PECARN ≥ 2 years**	**2018 (94·3%)**
Inclusion criteria	Age ≥ 2 years	2139 (71·0%)
Exclusion criteria	Pre-existing neurological disorders complicating assessment	83 (3·9%)
Exclusion criteria	Trivial injury mechanism	0 (0·0%)
Exclusion criteria	Bleeding disorder	5 (0·2%)
Exclusion criteria	GCS 9-13	22 (1·0%)
Exclusion criteria	Known brain tumours	0 (0·0%)
Exclusion criteria	Neuroimaging at an outside hospital before transfer	10 (0·9%)
Exclusion criteria	Ventricular shunts	0 (0·0%)
Exclusion criteria	Penetrating trauma*	0 (0·0%)

		Number of patients (%)
**Application rate**	**SNC16**	**3008 (99·9%)**
Inclusion criteria	Head trauma and age <18 years and <24 h from trauma and GCS 9-15 in ED	3012 (100·0%)
Exclusion criteria	Suspected non-accidental injury*	0 (0·0%)
Exclusion criteria	High-velocity injury mechanism	4 (0·1%)

		Number of patients (%)
**Application rate**	**PREDICT < 2 years**	**873 (100·0%)**
Inclusion criteria	Head trauma and age <2 years and <72 h from trauma** and GCS 9-15 in ED	873 (29·0%)

		Number of patients (%)
**Application rate**	**PREDICT ≥ 2 years**	**2139 (100·0%)**
Inclusion criteria	Head trauma and age ≥ 2 years and <72 h from trauma** and GCS 9-15 in ED	2139 (71·0%)

		Number of patients (%)
**Application rate**	**NICE23**	**2948 (97·3%)**
Inclusion criteria	Sustained a head injury	3012 (100·0%)
Exclusion criteria	Age ≥ 16 years	81 (2·7%)

§Minor head injury is defined as injury within the past 24 hours associated with witnessed loss of consciousness, definite amnesia, witnessed disorientation, persistent vomiting (more than one episode) or persistent irritability (in a child under two years of age) in a patient with a Glasgow Coma Scale score of 13–15, with age < 17 years.

*Exclusion criteria for the Scandinavian cohort

**Restricted to 24 hours from trauma due to inclusion criteria for the Scandinavian cohort

Supplementary Table S4. Diagnostic accuracy to predict neurosurgery in comparison cohort

	Sensitivity % (CI95)	Specificity % (CI95)	PPV % (CI95)	NPV % (CI95)
CHALICE	100·0% (15·8-100·0)	77·8% (76·3-79·3)	0·3% (0·0-1·1)	100·0% (99·8-100·0)
CATCH	100·0% (15·8-100·0)	74·0% (72·4-75·5)	0·3% (0·0-0·9)	100·0% (99·8-100·0)
CATCH2	100·0% (15·8-100·0)	70·2% (68·5-71·8)	0·2% (0·0-0·8)	100·0% (99·8-100·0)
PECARN < 2 years	100·0% (15·8-100·0)	55·0% (53·2-56·7)	0·1% (0·0-0·5)	100·0% (99·8-100·0)
PECARN ≥ 2 years	50·0% (1·3-98·7)	55·6% (53·8-57·4)	0·1% (0·0-0·4)	99·9% (99·7-100·0)
SNC16	100·0% (15·8-100·0)	41·2% (39·5-43·0)	0·1% (0·0-0·4)	100·0% (99·7-100·0)
PREDICT < 2 years	100·0% (15·8-100·0)	51·5% (49·7-53·3)	0·1% (0·0-0·5)	100·0% (99·8-100·0)
PREDICT ≥ 2 years	50·0% (1·3-98·7)	54·4% (52·6-56·1)	0·1% (0·0-0·4)	99·9% (99·7-100·0)
NICE23	100·0% (15·8-100·0)	76·9% (75·4-78·4)	0·3% (0·0-1·0)	100·0% (99·8-100·0)

Data presented when applying all risk predictors in respective guideline.

Supplementary Table S5. Diagnostic accuracy CATCH2

CATCH2				
**High risk predictors**				
**Application cohort (n=934/3012; 31·0%)**	**Sensitivity**	**Specificity**	**PPV**	**NPV**
Significant cCT (n=14)	64·3% (35·1-87·2)	77·5% (74·7-80·2)	4·2% (1·9-7·8)	99·3% (98·4-99·8)
Neurosurgery (n=0)	NA	76·9% (74·0-79·5)	0·0% (0·0-1·7)	100·0% (99·5-100·0)
Rule specific primary endpoint* (n=0)	NA	76·9% (74·0-79·5)	0·0% (0·0-1·7)	100·0% (99·5-100·0)
Rule specific secondary endpoint** (n=11)	54·5% (23·4-83·3)	77·2% (74·4-79·9)	2·8% (1·0-5·9)	99·3% (98·4-99·8)
**Comparison cohort (n=3012)**	**Sensitivity**	**Specificity**	**PPV**	**NPV**
Significant cCT (n=27)	48·1% (28·7-68·1)	90·7% (89·6-91·7)	4·5% (2·4-7·5)	99·5% (99·1-99·7)
Neurosurgery (n=2)	100·0% (15·8-100·0)	90·4% (89·3-91·4)	0·7% (0·1-2·5)	100·0% (99·9-100·0)
Rule specific primary endpoint* (n=3)	100·0% (29·2-100·0)	90·4% (89·3-91·4)	1·0% (0·2-3·0)	100·0% (99·9-100·0)
Rule specific secondary endpoint** (n=20)	50·0% (27·2-72·8)	90·6% (89·5-91·6)	3·4% (1·7-6·2)	99·6% (99·3-99·8)
**High and medium risk predictors**				
**Application cohort (n=934/3012; 31·0%)**	**Sensitivity**	**Specificity**	**PPV**	**NPV**
Significant cCT (n=14)	92·9% (66·1-99·8)	60·4% (57·2-63·6)	3·4% (1·8-5·8)	99·8% (99·0-100·0)
Neurosurgery (n=0)	NA	59·6% (56·4-62·8)	0·0% (0·0-1·0)	100·0% (99·3-100·0)
Rule specific primary endpoint* (n=0)	NA	59·6% (56·4-62·8)	0·0% (0·0-1·0)	100·0% (99·3-100·0)
Rule specific secondary endpoint** (n=11)	90·9% (58·7-99·8)	60·2% (57·0-63·4)	2·7% (1·3-4·8)	99·8% (99·0-100·0)
**CATCH2 CT-rate application cohort: 40·4% (CI95 37·3-43·5; 377/934)**				
**Comparison cohort (n=3012)**	**Sensitivity**	**Specificity**	**PPV**	**NPV**
Significant cCT (n=27)	85·2% (66·3-95·8)	70·6% (68·9-72·2)	2·6% (1·6-3·8)	99·8% (99·5-99·9)
Neurosurgery (n=2)	100·0% (15·8-100·0)	70·2% (68·5-71·8)	0·2% (0·0-0·8)	100·0% (99·8-100·0)
Rule specific primary endpoint* (n=3)	100·0% (29·2-100·0)	70·2% (68·5-71·8)	0·3% (0·1-1·0)	100·0% (99·8-100·0)
Rule specific secondary endpoint** (n=20)	85·0% (62·1-96·8)	70·5% (68·8-72·1)	1·9% (1·1-3·0)	99·9% (99·6-100·0)
**CATCH2 CT-rate comparison cohort: 29·9% (CI95 28·3-31·5; 900/3012)**				

*Need for neurological intervention defined as either death within 7 days secondary to the head injury or need for any of the following procedures within 7 days: craniotomy, elevation of skull fracture, monitoring of intracranial pressure, or insertion of an endotracheal tube for the management of head injury.

** TBI on CT defined as any acute intracranial finding revealed on CT that was attributable to acute injury, including closed depressed skull fracture (i.e., depressed past the inner table and pneumocephalus but excluding nondepressed skull fractures and basilar skull fractures)

Supplementary Table S6. Diagnostic accuracy CATCH

CATCH				
**High risk predictors**				
**Application cohort (n=934/3012; 31·0%)**	**Sensitivity**	**Specificity**	**PPV**	**NPV**
Significant cCT (n=14)	35·7% (12·8-64·9)	91·5% (89·5-93·2)	6·0% (2·0-13·5)	98·9% (98·0-99·5)
Neurosurgery (n=0)	NA	91·1% (89·1-92·9)	0·0% (0·0-4·3)	100·0% (99·6-100·0)
Rule specific primary endpoint* (n=0)	NA	91·1% (89·1-92·9)	0·0% (0·0-4·3)	100·0% (99·6-100·0)
Rule specific secondary endpoint** (n=11)	27·3% (6·0-61·0)	91·3% (89·3-93·1)	3·6% (0·8-10·2)	99·1% (98·2-99·6)
**Comparison cohort (n=3012)**	**Sensitivity**	**Specificity**	**PPV**	**NPV**
Significant cCT (n=27)	33·3% (16·5-54·0)	95·3% (94·5-96·0)	6·0% (2·8-11·1)	99·4% (99·0-99·6)
Neurosurgery (n=2)	100·0% (15·8-100·0)	95·1% (94·2-95·8)	1·3% (0·2-4·7)	100·0% (99·9-100·0)
Rule specific primary endpoint* (n=3)	100·0% (29·2-100·0)	95·1% (94·3-95·9)	2·0% (0·4-5·7)	100·0% (99·9-100·0)
Rule specific secondary endpoint** (n=20)	35·0% (15·4-59·2)	95·2% (94·4-96·0)	4·7% (1·9-9·4)	99·5% (99·2-99·8)
**High and medium risk predictors**				
**Application cohort (n=934/3012; 31·0%)**	**Sensitivity**	**Specificity**	**PPV**	**NPV**
Significant cCT (n=14)	78·6% (49·2-95·3)	71·8% (68·8-74·7)	4·1% (2·1-7·2)	99·5% (98·7-99·9)
Neurosurgery (n=0)	NA	71·1% (68·1-74·0)	0·0% (0·0-1·4)	100·0% (99·4-100·0)
Rule specific primary endpoint* (n=0)	NA	71·1% (68·1-74·0)	0·0% (0·0-1·4)	100·0% (99·4-100·0)
Rule specific secondary endpoint** (n=11)	72·7% (39·0-94·0)	71·6% (68·6-74·5)	3·0% (1·3-5·8)	99·5% (98·7-99·9)
**CATCH CT-rate application cohort: 28·9% (CI95 26·1-31·9; 270/934)**				
**Comparison cohort (n=3012)**	**Sensitivity**	**Specificity**	**PPV**	**NPV**
Significant cCT (n=27)	77·8% (57·7-91·4)	74·4% (72·8-76·0)	2·7% (1·7-4·1)	99·7% (99·4-99·9)
Neurosurgery (n=2)	100·0% (15·8-100·0)	74·0% (72·4-75·5)	0·3% (0·0-0·9)	100·0% (99·8-100·0)
Rule specific primary endpoint* (n=3)	100·0% (29·2-100·0)	74·0% (72·4-75·6)	0·4% (0·1-1·1)	100·0% (99·8-100·0)
Rule specific secondary endpoint** (n=20)	75·0% (50·9-91·3)	74·3% (72·7-75·8)	1·9% (1·1-3·1)	99·8% (99·5-99·9)
**CATCH CT-rate comparison cohort: 26·1% (CI95 24·5-27·7; 785/3012)**				

*Need for neurological intervention defined as either death within 7 days secondary to the head injury or need for any of the following procedures within 7 days: craniotomy, elevation of skull fracture, monitoring of intracranial pressure, or insertion of an endotracheal tube for the management of head injury.

** TBI on CT defined as any acute intracranial finding revealed on CT that was attributable to acute injury, including closed depressed skull fracture (i.e., depressed past the inner table and pneumocephalus but excluding nondepressed skull fractures and basilar skull fractures)

.

Supplementary Table S7. SNC16

SNC16				
**Application cohort (n=3008/3012; 99·9%)**	**Sensitivity**	**Specificity**	**PPV**	**NPV**
Significant cCT (n=27)	100·0% (87·2-100·0)	41·6% (39·8-43·4)	1·5% (1·0-2·2)	100·0% (99·7-100·0)
Neurosurgery (n=2)	100·0% (15·8-100·0)	41·3% (39·5-43·0)	0·1% (0·0-0·4)	100·0% (99·7-100·0)
Rule specific primary endpoint* (n=2)	100·0% (15·8-100·0)	41·3% (39·5-43·0)	0·1% (0·0-0·4)	100·0% (99·7-100·0)
Rule specific secondary endpoint** (n=26)	100·0% (86·8-100·0)	41·6% (39·8-43·4)	1·5% (1·0-2·1)	100·0% (99·7-100·0)
**SNC16 “mandatory CT” rate application cohort: 3·4% (CI95 2·8-4·0; 101/3008)**				
**SNC16 “CT or observation” rate application cohort: 19·5% (CI95 18·1-20·9; 586/3008)**				
**SNC16 “observation” rate application cohort: 35·9% (CI95 34·2-37·7; 1081/3008)**				
**SNC16 “any intervention” rate application cohort: 58·8% (CI95 57·0-60·5; 1768/3008)**				
**Comparison cohort (n=3012)**	**Sensitivity**	**Specificity**	**PPV**	**NPV**
Significant cCT (n=27)	100·0% (87·2-100·0)	41·6% (39·8-43·4)	1·5% (1·0-2·2)	100·0% (99·7-100·0)
Neurosurgery (n=2)	100·0% (15·8-100·0)	41·2% (39·5-43·0)	0·1% (0·0-0·4)	100·0% (99·7-100·0)
Rule specific primary endpoint* (n=2)	100·0% (15·8-100·0)	41·2% (39·5-43·0)	0·1% (0·0-0·4)	100·0% (99·7-100·0)
Rule specific secondary endpoint** (n=26)	100·0% (86·8-100·0)	41·6% (39·8-43·4)	1·5% (1·0-2·1)	100·0% (99·7-100·0)
**SNC16 “mandatory CT-rate” comparison cohort: 3·4% (CI95 2·8-4·0; 101/3012)**				
**SNC16 “CT or observation” rate comparison cohort: 19·5% (CI95 18·1-21·0; 588/3012)**				
**SNC16 “observation” rate comparison cohort: 35·9% (CI95 34·2-37·6; 1082/3012)**				
**SNC16 “any intervention” rate comparison cohort: 58·8% (CI95 57·0-60·5; 1771/3012)**				

*Need for neurosurgical intervention defined as any neurosurgical procedure for cranial or intracranial injury within the first week following trauma, but also neurointensive care measures.

** Any traumatic intracranial injury (ICI). Intracranial injury (ICI) is any intracranial pathology on head CT, such as intracerebral haematomas, epidural and subdural haematomas, traumatic subarachnoid haemorrhage, pneumocephalus, depressed skull fracture and presence of skull base fracture, except isolated linear non-depressed skull fractures.

Supplementary Table S8. CHALICE

CHALICE				
**Application cohort (n=2948/3012; 97·9%)**	**Sensitivity**	**Specificity**	**PPV**	**NPV**
Significant cCT (n=25)	84·0% (63·9-95·5)	78·3% (76·7-79·8)	3·2% (2·0-4·9)	99·8% (99·6-100·0)
Neurosurgery (n=2)	100·0% (15·8-100·0)	77·8% (76·3-79·3)	0·3% (0·0-1·1)	100·0% (99·8-100·0)
Rule specific primary endpoint* (n=24)	83·3% (62·6-95·3)	78·2% (76·7-79·7)	3·0% (1·9-4·7)	99·8% (99·6-100·0)
Rule specific secondary endpoint** (n=503)	50·5% (46·0-55·0)	83·6% (82·0-85·0)	38·7% (35·0-42·6)	89·1% (87·8-90·4)
**CHALICE CT-rate application cohort: 22·3% (CI95 20·8-23·8; 656/2948)**				
**Comparison cohort (n=3012)**	**Sensitivity**	**Specificity**	**PPV**	**NPV**
Significant cCT (n=27)	81·5% (61·9-93·7)	78·3% (76·8-79·8)	3·3% (2·1-4·9)	99·8% (99·5-99·9)
Neurosurgery (n=2)	100·0% (15·8-100·0)	77·8% (76·3-79·3)	0·3% (0·0-1·1)	100·0% (99·8-100·0)
Rule specific primary endpoint* (n=26)	80·8% (60·6-93·4)	78·3% (76·8-79·8)	3·1% (2·0-4·8)	99·8% (99·5-99·9)
Rule specific secondary endpoint** (n=517)	50·1% (45·7-54·5)	83·6% (82·1-85·0)	38·7% (35·0-42·5)	89·0% (87·7-90·2)
**CHALICE CT-rate comparison cohort: 22·2% (CI95 20·8-23·7; 669/3012)**				

*Clinically significant intracranial injury defined as death as a result of head injury, requirement for neurosurgical intervention, or marked abnormality on CT defined as any new, acute, traumatic intracranial pathology as reported by consultant radiologist, including intracranial haematomas of any size, cerebral contusion, diffuse cerebral oedema, and depressed skull fracture.

** Presence of skull fracture or admission to hospital

Supplementary Table S9. PECARN < 2 years

PECARN <2 y				
ALL RISK FACTORS				
**Application cohort (n=861/873; 98·6%)**	**Sensitivity**	**Specificity**	**PPV**	**NPV**
Significant cCT (n=9)	100·0% (66·4-100·0)	54·9% (51·5-58·3)	2·3% (1·1-4·3)	100·0% (99·2-100·0)
Neurosurgery (n=0)	NA	54·4% (51·0-57·7)	0·0% (0·0-0·9)	100·0% (99·2-100·0)
Rule specific primary endpoint* (n=1)	100·0% (2·5-100·0)	54·4% (51·0-57·8)	0·3% (0·0-1·4)	100·0% (99·2-100·0)
**PECARN<2y CT-rate in application cohort: 4·4% (CI95 3·2-5·9; 38/861)**				
**PECARN <2y “CT or observation”-rate in application cohort: 41·2% (CI95 38·0-44·5; 355/861)**				
**PECARN <2y “any intervention”-rate in application cohort: 45·6% (CI95 42·3-49·0; 393/861)**				
**Comparison cohort (n=3012)**	**Sensitivity**	**Specificity**	**PPV**	**NPV**
Significant cCT (n=27)	85·2% (66·3-95·8)	55·3% (53·5-57·1)	1·7% (1·1-2·5)	99·8% (99·4-99·9)
Neurosurgery (n=2)	100·0% (15·8-100·0)	55·0% (53·2-56·7)	0·1% (0·0-0·5)	100·0% (99·8-100·0)
Rule specific primary endpoint* (n=6)	100·0% (54·1-100·0)	55·0% (53·2-56·8)	0·4% (0·2-1·0)	100·0% (99·8-100·0)
**PECARN <2y CT-rate in comparison cohort: 5·7% (CI95 4·9-6·6; 172/3012)**				
**PECARN <2y “CT or observation”-rate in comparison cohort: 39·4% (CI95 37·6-41·1; 1186/3012)**				
**PECARN <2y “any intervention”-rate in comparison cohort: 45·1% (CI95 43·3-46·9; 1358/3012)**				
**HIGH-RISK FACTORS**				
**Application cohort (n=861/873; 98·6%)**	**Sensitivity**	**Specificity**	**PPV**	**NPV**
Significant cCT (n=9)	77·8% (40·0-97·2)	96·4% (94·9-97·5)	18·4% (7·7-34·3)	99·8% (99·1-100·0)
Neurosurgery (n=0)	NA	95·6% (94·0-96·9)	0·0% (0·0-9·3)	100·0% (99·6-100·0)
Rule specific primary endpoint* (n=1)	100·0% (2·5-100·0)	95·7% (94·1-97·0)	2·6% (0·1-13·8)	100·0% (99·6-100·0)
**Comparison cohort (n=3012)**	**Sensitivity**	**Specificity**	**PPV**	**NPV**
Significant cCT (n=27)	59·3% (38·8-77·6)	94·8% (93·9-95·5)	9·3% (5·4-14·7)	99·6% (99·3-99·8)
Neurosurgery (n=2)	100·0% (15·8-100·0)	94·4% (93·5-95·1)	1·2% (0·1-4·1)	100·0% (99·9-100·0)
Rule specific primary endpoint* (n=6)	66·7% (22·3-95·7)	94·4% (93·5-95·2)	2·3% (0·6-5·8)	99·9% (99·7-100·0)

*Clinically important TBI defined as death from TBI, neurosurgical intervention for TBI (intracranial pressure monitoring, elevation of depressed skull fracture, ventriculostomy, haematoma evacuation, lobectomy, tissue debridement, dura repair, or other), intubation of more than 24 h for TBI or hospital admission of 2 nights or more for TBI‡ in association with TBI on CT defined by any of the following descriptions: intracranial haemorrhage or contusion, cerebral oedema, traumatic infarction, diffuse axonal injury, shearing injury, sigmoid sinus thrombosis, midline shift of intracranial contents or signs of brain herniation, diastasis of the skull, pneumocephalus, skull fracture depressed by at least the width of the table of the skull.

Supplementary Table S10. PECARN ≥ 2 years

PECARN 2-17 y				
**ALL RISK FACTORS**				
**Application cohort (n=2018/2139; 94·3%)**	**Sensitivity**	**Specificity**	**PPV**	**NPV**
Significant cCT (n=14)	85·7% (57·2-98·2)	54·4% (52·2-56·6)	1·3% (0·7-2·3)	99·8% (99·3-100·0)
Neurosurgery (n=2)	50·0% (1·3-98·7)	54·2% (52·0-56·4)	0·1% (0·0-0·6)	99·9% (99·5-100·0)
Rule specific primary endpoint* (n=5)	80·0% (28·4-99·5)	54·2% (52·0-56·4)	0·4% (0·1-1·1)	99·9% (99·5-100·0)
**PECARN 2-17 years CT-rate in application cohort: 4·7% (CI95 3·8-5·6; 94/2018)**				
**PECARN 2-17 years "CT or observation"-rate in application cohort: 41·2% (CI95 39·0-43·3; 831/2018)**				
**PECARN 2-17 years "any intervention"-rate in application cohort: 45·8% (CI95 43·7-48·0; 925/2018)**				
**Comparison cohort (n=3012)**	**Sensitivity**	**Specificity**	**PPV**	**NPV**
Significant cCT (n=27)	74·1% (53·7-88·9)	55·8% (54·0-57·6)	1·5% (0·9-2·3)	99·6% (99·1-99·8)
Neurosurgery (n=2)	50·0% (1·3-98·7)	55·6% (53·8-57·4)	0·1% (0·0-0·4)	99·9% (99·7-100·0)
Rule specific primary endpoint* (n=6)	66·7% (22·3-95·7)	55·6% (53·8-57·4)	0·3% (0·1-0·8)	99·9% (99·6-100·0)
**PECARN 2-17 years CT-rate in comparison cohort: 5·3% (CI95 4·5-6·1; n=159/3012)**				
**PECARN 2-17 years "CT or observation"-rate comparison cohort: 39·1% (CI95 37·4-40·9; 1179/3012)**				
**PECARN 2-17 years "any intervention"-rate comparison cohort: 44·4% (CI95 42·7-46·2; 1338/3012)**				
**HIGH-RISK FACTORS**				
**Application cohort (n=2018/2139; 94·3%)**	**Sensitivity**	**Specificity**	**PPV**	**NPV**
Significant cCT (n=14)	42·9% (17·7-71·1)	95·6% (94·6-96·5)	6·4% (2·4-13·4)	99·6% (99·2-99·8)
Neurosurgery (n=2)	50·0% (1·3-98·7)	95·4% (94·4-96·3)	1·1% (0·0-5·8)	99·9% (99·7-100·0)
Rule specific primary endpoint* (n=5)	60·0% (14·7-94·7)	95·5% (94·5-96·3)	3·2% (0·7-9·0)	99·9% (99·6-100·0)
**Comparison cohort (n=3012)**	**Sensitivity**	**Specificity**	**PPV**	**NPV**
Significant cCT (n=27)	44·4% (25·5-64·7)	95·1% (94·2-95·8)	7·5% (4·0-12·8)	99·5% (99·1-99·7)
Neurosurgery (n=2)	50·0% (1·3-98·7)	94·8% (93·9-95·5)	0·6% (0·0-3·5)	100·0% (99·8-100·0)
Rule specific primary endpoint* (n=6)	50·0% (11·8-88·2)	94·8% (94·0-95·6)	1·9% (0·4-5·4)	99·9% (99·7-100·0)

*Clinically important TBI defined as death from TBI, neurosurgical intervention for TBI (intracranial pressure monitoring, elevation of depressed skull fracture, ventriculostomy, haematoma evacuation, lobectomy, tissue debridement, dura repair, or other), intubation of more than 24 h for TBI or hospital admission of 2 nights or more for TBI‡ in association with TBI on CT defined by any of the following descriptions: intracranial haemorrhage or contusion, cerebral oedema, traumatic infarction, diffuse axonal injury, shearing injury, sigmoid sinus thrombosis, midline shift of intracranial contents or signs of brain herniation, diastasis of the skull, pneumocephalus, skull fracture depressed by at least the width of the table of the skull.

Supplementary Table S11. PREDICT < 2 years

PREDICT <2 y				
**Application cohort (n=873/873; 100·0%)**	**Sensitivity**	**Specificity**	**PPV**	**NPV**
Significant cCT (n=9)	100·0% (66·4-100·0)	45·9% (42·6-49·3)	1·9% (0·9-3·6)	100·0% (99·1-100·0)
Neurosurgery (n=0)	NA	45·5% (42·1-48·8)	0·0% (0·0-0·8)	100·0% (99·1-100·0)
Rule specific primary endpoint – PREDICT* (n=1)	100·0% (2·5-100·0)	45·5% (42·2-48·9)	0·2% (0·0-1·2)	100·0% (99·1-100·0)
**PREDICT mandatory CT-rate application cohort: 1·7% (CI95 1·0-2·7; 15/873)**				
**PREDICT Optional "CT or observation" rate application cohort: 13·6% (CI95 11·5-16·0; 119/873)**				
**PREDICT observation rate application cohort: 39·2% (CI95 36·0-42·4; 342/873)**				
**PREDICT "any intervention" rate application cohort: 54·5% (CI95 51·2-57·8; 476/873)**				
**Comparison cohort (n=3012)**	**Sensitivity**	**Specificity**	**PPV**	**NPV**
Significant cCT (n=27)	85·2% (66·3-95·8)	51·8% (50·0-53·6)	1·6% (1·0-2·4)	99·7% (99·3-99·9)
Neurosurgery (n=2)	100·0% (15·8-100·0)	51·5% (49·7-53·3)	0·1% (0·0-0·5)	100·0% (99·8-100·0)
Rule specific primary endpoint – PREDICT* (n=5)	80·0% (28·4-99·5)	51·5% (49·7-53·3)	0·3% (0·1-0·7)	99·9% (99·6-100·0)
**PREDICT mandatory CT-rate comparison cohort: 1·7% (CI95 1·2-2·2; 50/3012)**				
**PREDICT Optional "CT or observation" rate comparison cohort: 13·2% (CI95 12·0-14·5; 398/3012)**				
**PREDICT observation rate comparison cohort: 33·6% (CI95 32·0-35·3; 1013/3012)**				
**PREDICT "any intervention" rate comparison cohort: 48·5% (CI95 46·7-50·3; 1461/3012)**				

*Clinically important intracranial injury in need for intervention (as neurosurgery and/or intensive care)

Supplementary Table S12. PREDICT ≥ 2 years

PREDICT ≥2 y				
**Application cohort (n=2139/2139; 100·0%)**	**Sensitivity**	**Specificity**	**PPV**	**NPV**
Significant cCT (n=18)	88·9% (65·3-98·6)	52·2% (50·0-54·3)	1·6% (0·9-2·5)	99·8% (99·4-100·0)
Neurosurgery (n=2)	50·0% (1·3-98·7)	51·8% (49·7-54·0)	0·1% (0·0-0·5)	99·9% (99·5-100·0)
Rule specific primary endpoint – PREDICT* (n=4)	75·0% (19·4-99·4)	51·9% (49·8-54·0)	0·3% (0·1-0·8)	99·9% (99·5-100·0)
**PREDICT mandatory CT-rate application cohort: 1·5% (CI95 1·1-2·1; 33/2139)**				
**PREDICT Optional "CT or observation" rate application cohort: 9·8% (CI95 8·6-11·1; 209/2139)**				
**PREDICT observation rate application cohort: 36·8% (CI95 34·8-38·9; 788/2139)**				
**PREDICT "any intervention" rate application cohort: 48·2% (CI95 46·0-50·3; 1030/2139)**				
**Comparison cohort (n=3012)**	**Sensitivity**	**Specificity**	**PPV**	**NPV**
Significant cCT (n=27)	74·1% (53·7-88·9)	54·6% (52·8-56·4)	1·5% (0·9-2·2)	99·6% (99·1-99·8)
Neurosurgery (n=2)	50·0% (1·3-98·7)	54·4% (52·6-56·1)	0·1% (0·0-0·4)	99·9% (99·7-100·0)
Rule specific primary endpoint – PREDICT* (n=5)	80·0% (28·4-99·5)	54·4% (52·6-56·2)	0·3% (0·1-0·7)	99·9% (99·7-100·0)
**PREDICT mandatory CT-rate comparison cohort: 1·2% (CI95 0·8-1·6; 35/3012)**				
**PREDICT Optional "CT or observation" rate comparison cohort: 8·4% (CI95 7·4-9·4; 253/3012)**				
**PREDICT observation rate comparison cohort: 36·1% (CI95 34·4-37·8; 1087/3012)**				
**PREDICT "any intervention" rate comparison cohort: 45·7% (CI95 43·9-47·4; 1375/3012)**				

*Clinically important intracranial injury in need for intervention (as neurosurgery and/or intensive care)

Supplementary Table S13. NICE23

NICE23				
**Application cohort (n=2948/3012; 97·9%)**	**Sensitivity**	**Specificity**	**PPV**	**NPV**
Significant cCT (n=25)	84·0% (63·9-95·5)	77·4% (75·8-78·9)	3·1% (1·9-4·7)	99·8% (99·5-100·0)
Neurosurgery (n=2)	100·0% (15·8-100·0)	76·9% (75·3-78·4)	0·3% (0·0-1·1)	100·0% (99·8-100·0)
Rule specific primary endpoint - CHALICE (n=24) #	83·3% (62·6-95·3)	77·3% (75·8-78·8)	2·9% (1·8-4·5)	99·8% (99·5-100·0)
Rule specific secondary endpoint - CHALICE (n=503) #	51·9% (47·4-56·3)	82·7% (81·2-84·2)	38·2% (34·5-42·0)	89·3% (88·0-90·6)
**NICE23 mandatory CT-rate* application cohort: 8·4% (CI95 7·5-9·5; 249/2948)**				
**NICE23 observation rate** application cohort: 14·7% (CI95 13·5-16·0; 434/2948)**				
**NICE23 CT or observation rate*** application cohort: 23·2% (CI95 21·7-24·7; 683/2948)**				
**Comparison cohort (n=3012)**	**Sensitivity**	**Specificity**	**PPV**	**NPV**
Significant cCT (n=27)	81·5% (61·9-93·7)	77·4% (75·9-78·9)	3·2% (2·0-4·7)	99·8% (99·5-99·9)
Neurosurgery (n=2)	100·0% (15·8-100·0)	76·9% (75·4-78·4)	0·3% (0·0-1·0)	100·0% (99·8-100·0)
Rule specific primary endpoint - CHALICE (n=26) #	80·8% (60·6-93·4)	77·4% (75·9-78·9)	3·0% (1·9-4·6)	99·8% (99·5-99·9)
Rule specific secondary endpoint - CHALICE (n=517) #	51·5% (47·0-55·8)	82·8% (81·2-84·2)	38·2% (34·6-41·9)	89·2% (87·8-90·4)
**NICE23 "mandatory CT"-rate* comparison cohort: 8·4% (CI95 7·4-9·4; 252/3012)**				
**NICE23 "observation" rate** comparison cohort: 14·7% (CI95 13·5-16·; 444/3012)**				
**NICE23 "any intervention" rate*** comparison cohort: 23·1% (CI95 21·6-24·6; 696/3012)**				

*Presence of one or more high-risk factor, or 2 or more low-risk factors

** Presence of one low-risk factor, in the absence of multiple low-risk factors or high-risk factor (s)

*** Presence of at least one risk factor (high or/and low risk).

Supplementary Table S14. Diagnostic accuracy to predict rule-specific primary endpoint in application cohorts

	Sensitivity<br>% (CI95)	Specificity<br>% (CI95)	PPV<br>% (CI95)	NPV<br>% (CI95)
CHALICE (n=24)	83·3% (62·6-95·3)	78·2% (76·7-79·7)	3·0% (1·9-4·7)	99·8% (99·6-100·0)
CATCH (n=0)	NA	71·1% (68·1-74·0)	0·0% (0·0-1·4)	100·0% (99·4-100·0)
CATCH2 (n=0)	NA	59·6% (56·4-62·8)	0·0% (0·0-1·0)	100·0% (99·3-100·0)
PECARN < 2 years (n=1)	100·0% (2·5-100·0)	54·4% (51·0-57·8)	0·3% (0·0-1·4)	100·0% (99·2-100·0)
PECARN 2-17 years (n=5)	80·0% (28·4-99·5)	54·2% (52·0-56·4)	0·4% (0·1-1·1)	99·9% (99·5-100·0)
SNC16 (n=2)	100·0% (15·8-100·0)	41·3% (39·5-43·0)	0·1% (0·0-0·4)	100·0% (99·7-100·0)
PREDICT < 2 years (n=1)	100·0% (2·5-100·0)	45·5% (42·2-48·9)	0·2% (0·0-1·2)	100·0% (99·1-100·0)
PREDICT ≥ 2 years (n=4)	75·0% (19·4-99·4)	51·9% (49·8-54·0)	0·3% (0·1-0·8)	99·9% (99·5-100·0)
NICE23<br>(primary endpoint CHALICE; n=24)	83·3% (62·6-95·3)	77·3% (75·8-78·8)	2·9% (1·8-4·5)	99·8% (99·5-100·0)

Data presented when applying all risk predictors in respective guideline.

Supplementary Table S15. CT and observation rates in the application cohorts

	Mandatory CT<br>% (CI95)	CT or observation<br>% (CI95)	Observation<br>% (CI95)	Sum (any intervention)<br>% (CI95)
CHALICE	22·3% (20·8-23·8)			22·3% (20·8-23·8)
CATCH	28·9% (26·1-31·9)			28·9% (26·1-31·9)
CATCH2	40·4% (37·3-43·5)			40·4% (37·3-43·5)
PECARN < 2 years	4·4% (3·2-5·9)	41·2% (38·0-44·5)		45·6% (42·3-49·0)
PECARN 2-17 years	4·7% (3·8-5·6)	41·2% (39·0-43·3)		45·8% (43·7-48·0)
SNC16	3·4% (2·8-4·0)	19·5% (18·1-20·9)	35·9% (34·2-37·7)	58·8% (57·0-60·5)
PREDICT < 2 years	1·7% (1·0-2·7)	13·6% (11·5-16·0)	39·2% (36·0-42·4)	54·5% (51·2-57·8)
PREDICT ≥ 2 years	1·5% (1·1-2·1)	9·8% (8·6-11·1)	36·8% (34·8-38·9)	48·2% (46·0-50·3)
NICE23	8·4% (7·5-9·5)		14·7% (13·5-16·0)	23·2% (21·7-24·7)

Data presented when applying all risk predictors in respective guideline.
